# Transcriptomic and metabolomic analyses of *Lycium ruthenicum* and *Lycium barbarum* fruits during ripening

**DOI:** 10.1038/s41598-020-61064-5

**Published:** 2020-03-09

**Authors:** Jianhua Zhao, Haoxia Li, Yue Yin, Wei An, Xiaoya Qin, Yajun Wang, Yanlong Li, Yunfang Fan, Youlong Cao

**Affiliations:** 1 Wolfberry Engineering Research Institute, Ningxia Academy of Agriculture and Forestry Sciences/National Wolfberry Engineering Research Center, Yinchuan, 750002 China; 2 Desertification Control Research Institute, Ningxia Academy of Agriculture and Forestry Sciences, Yinchuan, Ningxia 750002 China

**Keywords:** Gene expression, Gene expression, Transcriptomics, Transcriptomics

## Abstract

Red wolfberry (or goji berry, *Lycium barbarum*; LB) is an important agricultural product with a high content of pharmacologically important secondary metabolites such as phenylpropanoids. A close relative, black wolfberry (*L. ruthenicum*; LR), endemic to the salinized deserts of northwestern China, is used only locally. The two fruits exhibit many morphological and phytochemical differences, but genetic mechanisms underlying them remain poorly explored. In order to identify the genes of interest for further studies, we studied transcriptomic (Illumina HiSeq) and metabolomic (LC-MS) profiles of the two fruits during five developmental stages (young to ripe). As expected, we identified much higher numbers of significantly differentially regulated genes (DEGs) than metabolites. The highest numbers were identified in pairwise comparisons including the first stage for both species, but total numbers were consistently somewhat lower for the LR. The number of differentially regulated metabolites in pairwise comparisons of developmental stages varied from 66 (stages 3 vs 4) to 133 (stages 2 vs 5) in both species. We identified a number of genes (e.g. *AAT1*, *metE*, *pip*) and metabolites (e.g. rutin, raffinose, galactinol, trehalose, citrulline and DL-arginine) that may be of interest to future functional studies of stress adaptation in plants. As LB is also highly suitable for combating soil desertification and alleviating soil salinity/alkalinity/pollution, its potential for human use may be much wider than its current, highly localized, relevance.

## Introduction

Goji berry (also known as red wolfberry), used in China as food and medicine for millennia, recently achieved almost global popularity due to it being advertised as a healthy, ageing-preventing food. In the traditional Chinese medicine, goji is traditionally consumed for its alleged anti-aging, tranquilizing and Yin strengthening properties^1,2^. Although the clinical efficacy remains to be fully confirmed, there is some evidence that goji extracts may be beneficial for the prevention and treatment of age-related disorders, diabetes, hyperlipidaemia, cancer, hepatitis, immune disorders, thrombosis, and male infertility^1,3^. Specifically, *Lycium* fruits have relatively high content of bioactive components believed to be pharmacologically important, e.g. possessing immuno-enhancement and antioxidative activities, such as polyphenols, phenylpropanoids, carotenoids and polysaccharides^3–6^.

Two closely related species are sometimes sold as goji berries, *Lycium barbarum* and *L. chinense* (Chinese boxthorn) but nearly 90% of all commercially available goji berries belong to the former species^2^. Although the native range of this species is probably in the Mediterranean Basin, a majority of global commercial production takes place in arid and semi-arid areas of two provinces in Northwest China, Ningxia and Xinjiang^1^. *Lycium ruthenicum* (Russian box thorn or black wolfberry), a very close relative of *L. barbarum* and *L. chinense*^7^, is a wild perennial thorny shrub native to Northwest China, whose resistance to the harsh environment of saline deserts makes it a popular choice plant for combating soil desertification and for alleviating soil salinity/alkalinity^8,9^. It is also used in the local folk medicine and as food^8^, and studies indicate that it has notable pharmaceutical effects^9,10^. As these two species, and their fruits, are referred to by a range of (often overlapping) names^1^, to avoid confusion we refer to the fruit of *L. barbarum* (LB) as red wolfberry, and fruit of *L. ruthenicum* (LR) as black wolfberry. Despite their close phylogenetic relationship, the fruits of these two species exhibit distinct phenotypic profiles of during all developmental stages, including their shape, size, colour, taste, nutritional value and pharmacological properties^3,4,11^. As opposed to the red and elongated mature red wolfberry, black wolfberry is dark-purple or black, round, and smaller. It is also known that metabolic phenotypes of these two fruits differ significantly, particularly in the content of fatty acids, phenols and antioxidant capacities, which are much higher in black wolfberry, while the content of carotenoids, sugars, amino acids and osmolytes is higher in the red wolfberry^3,4^.

Fruit ripening is a complex developmental process, coordinated by a network of interacting genes and signalling pathways^12^, so genetic mechanisms underpinning these phenotypic differences remain only partially understood. The objective of this study was to contribute to our understanding of the complexity of ripening processes of these two fruits in different environments. To achieve this, we collected *L. barbarum* and *L. ruthenicum* fruits at five developmental stages, from young to ripe fruit, and sequenced their transcriptomes and metabolomes. These data shall help us better understand genetic underpinnings of both within- and between-species phenotypic differences that fruits of these two species exhibit during their respective ripening processes.

## Materials and methods

### Sample collection

**Figure 1:**
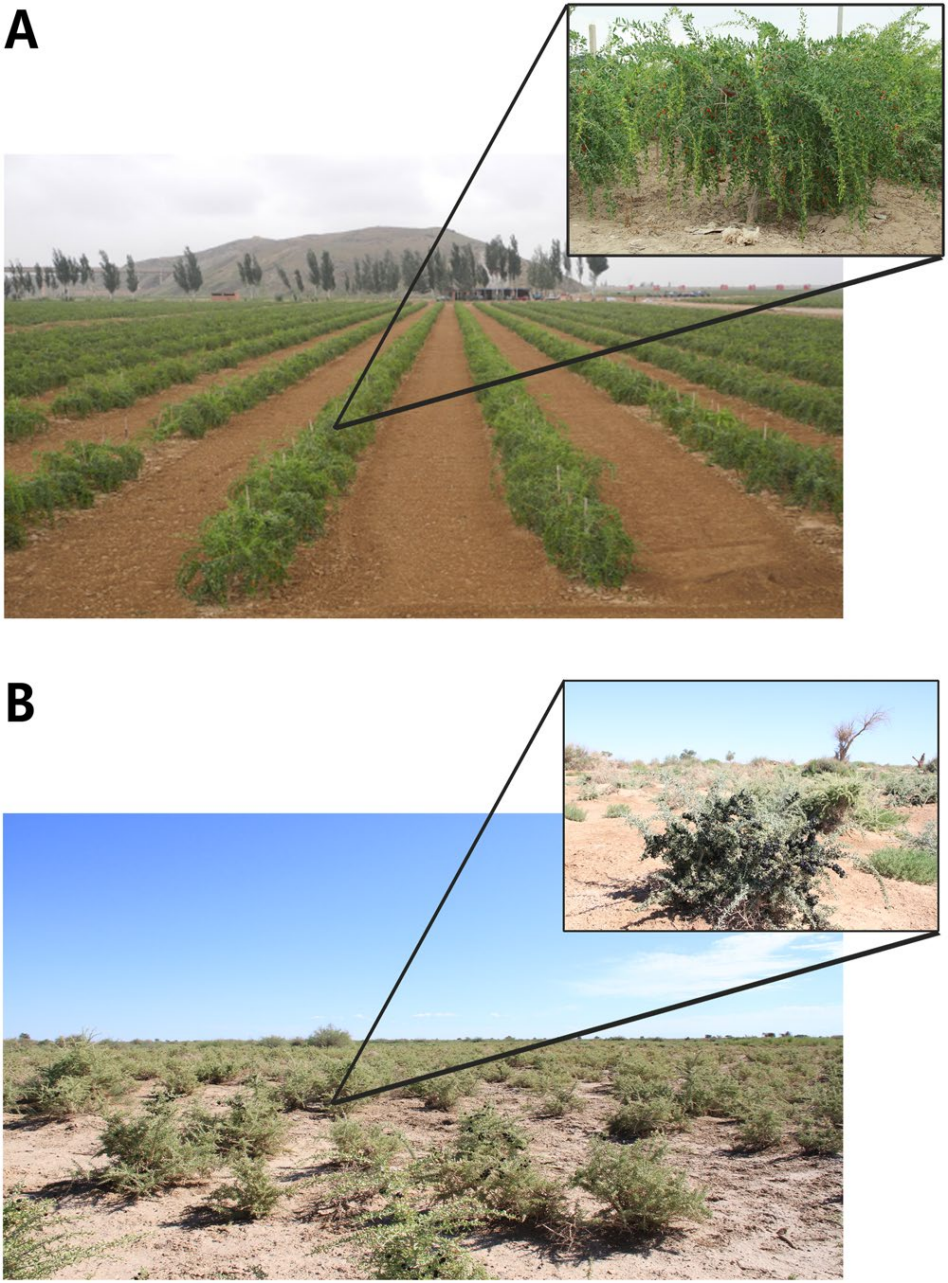
The studied species and sampling localities. (**A**) Cultured *Lycium barbarum* and (**B**) wild *Lycium ruthenicum* shrubs growing at the sampling locations.

Fruits were collected between July 1^st^ and August 20^th^ 2017 from nine wild *L. ruthenicum* (LR) shrubs growing in the vicinity of Bayan Taolaisu Wooden, Ejina, Alxa, Inner Mongolia, China (38 °38′49″ N; 106 °91′10″E; elevation = 1162 m) and nine 5-year-old cultured *L. barbarum* (LB) shrubs from the germplasm nursery of the Ningxia Academy of Agriculture and Forestry Science, Lu Hua Tai plantations, Xixia District, Yinchuan, Ningxia, China (41 °84′86″N; 100 °97′69″E; elevation = 948 m) (Fig. 1). Environmental characteristics of the two locations are (respectively): the average annual rainfall is <40 and <150 mm, the average temperature in July and August is 26.3 and 23.4 °C, soil types are salinized meadow and light sierozem, and surface salinity is 1.11% and 0.09%. The LB shrubs were regularly watered, so they did not undergo a major drought stress. As the two species have slightly different and variable fruit ripening periods, to be able to compare different ripening stages, we roughly divided the ripening period into five stages, and collected samples in the following time-windows after the flowering (anthesis): S1 - young fruit (9–12 days); S2 - green fruit (14–19 days); S3 - colouring fruit (20–26 days); S4 - immature fruit (30–37 days); S5 - mature fruit (34–45 days). Each sampling was conducted in the morning between 9am and 10am, from the south-facing side of the tree, approximately from the same spot on the same branch. At each sampling time-point several fruits were collected from three trees of each species. The three trees represented biological replicates in the transcriptome analysis. All samples were frozen immediately in liquid nitrogen and stored at −80 °C for further use.

### Transcriptome analysis

#### RNA extraction and sequencing

All fruit samples were ground to a powder in liquid nitrogen, and total RNA was extracted using MiniBEST Universal RNA Extraction Kit (Takara, Dalian, China). The extracted total RNA was treated with RQ1 DNase (Promega), and its quality and quantity then determined by measuring the absorbance at A260/A280 and A260/A230 using Smartspec plus (BioRad) spectrophotometer. RNA integrity was further verified by agarose gel electrophoresis. For each sample, 10 μg of total RNA was used for RNA-seq library preparation. Polyadenylated mRNAs were purified and concentrated with oligo (dT)-conjugated magnetic beads (Invitrogen) before being used for directional RNA-seq library preparation. The cDNA libraries were prepared from the purified mRNAs using the TruSeq Stranded Total RNA LT Sample Prep Kit (Illumina, CA, USA). Briefly: purified mRNAs were iron-fragmented at 95 °C, followed by end repair and 5′ adaptor ligation. Reverse transcription was then performed with RT primer included in the kit, harbouring a 3′ adaptor sequence and a randomized hexamer. The cDNAs were purified and amplified, and PCR products in the range 200–500 bp were purified, quantified and stored at −80 °C until the sequencing step. For high-throughput sequencing, the libraries were prepared following the manufacturer’s instructions and Illumina HiSeq. 2000 system used for 150 nt single-end sequencing.

#### Gene annotation

Raw reads were filtered using Cutadapt 1.7.1^13^: firstly we discarded all reads containing more than two N bases, then reads were processed by clipping the adaptor, removing low quality bases, and discarding short (<16 nt) reads. Uniquely localized clean reads were used to analyse the data quality with dupRadar^14^ with default parameters, and to calculate the read number and FPKM value (fragments per kilobase of transcripts per million mapped fragments) for each unigene according to reads and their genomic location^15^. *De novo* transcriptome assembly was conducted using Trinity program^16^, with default settings. The assembled transcriptomes were clustered using Corset^17^, a software designed for obtaining gene-level counts from any *de novo* transcriptome assembly. Unigenes were annotated by querying them against several public databases: Nr (NCBI non-redundant protein sequences), Nt (NCBI non-redundant nucleotide sequences), Pfam (Protein family)^18^, KOG/COG (Clusters of Orthologous Groups of proteins)^19,20^, Swiss-Prot (manually annotated and reviewed protein sequences)^21^, KO (KEGG Ontology)^22^, and GO (Gene Ontology)^23^. To further analyse the metabolic pathways different between the two sets of samples, all unigenes were queried against the KEGG pathway database. All BLASTx^24^ searches were performed with the e-value of 1E^**−**5^.

#### Differentially expressed genes (DEGs)

As our samples represented a time-series experiment, to explore temporal profiles of DEGs during the fruit development, we analysed the gene expression patterns using a time-series analysis tool maSigPro, an R package designed for identification of significantly different temporal expression profiles in RNA-seq data and microarray experiments^25^. The fold–changes in gene expression were also estimated with this package, and False Discovery Rate (FDR) thresholds were set to 0.01 and 0.7 R^2^. After obtaining the expression level of all genes in all of the samples, differentially expressed genes (DEGs) between the two fruits in the same developmental stages were analysed using Edge R^26^ with TMM normalization^27^. The following parameters were used to set the threshold for identifying DEGs: FDR < 0.01 and |fold change| ≥ 2.0. For each gene, the p-value was obtained on the basis of the model of negative binomial distribution. Benjamini-Hochberg procedure^28^ was used to control the false discovery rate (FDR) and infer the q-value (an adjusted p-value, taking in to account the FDR).

### Functional analysis of differentially expressed genes (DEGs)

Cluster analysis of gene expression patterns was performed with Cluster^29^ and Java Treeview^30^ software programs. To predict gene functions and calculate the functional category distribution frequency, KEGG analyses were employed using DAVID bioinformatics resources^31^. Functional networks were constructed by calculating the Pearson correlation coefficient (PCC) of the DEGs, and Cytoscape 3.0.2 was used to display the co-expression network^32^. Reliability of the RNA-seq data was corroborated by studying the expression of five randomly selected DEGs by qPCR (all details are provided in the Supplementary file: Supplementary Results).

### Metabolome analyses

Five fruit samples were used for each stage (5 biological replicates). About 100 mg of fruit tissue was crushed using a mixer mill (MM 400, Retsch) with a zirconia bead for 1.5 min at 30 Hz and extracted overnight at 4 °C with 0.6 ml 70% aqueous methanol. Following centrifugation at 10,000 g for 10 min, the extracts were absorbed (CNWBOND Carbon-GCB SPE Cartridge, 250 mg, 3 ml; ANPEL, Shanghai, China) and filtrated (SCAA-104, 0.22 μm pore size; ANPEL) before LCMS analysis. The sample extracts were analysed using an LC-ESI-MS/MS system (HPLC, Shim-pack UFLC SHIMADZU CBM30A system; MS, Applied Biosystems 4500 Q TRAP). The analytical conditions were as follows: HPLC column, Waters ACQUITY UPLC HSS T3 C18 (1.8 µm, 2.1 mm × 100 mm); and the mobile phase consisted of solvent A (pure water with 0.04% acetic acid) and solvent B (acetonitrile with 0.04% acetic acid). Sample measurements were performed with a gradient program that employed the starting conditions of 95% of solvent A and 5% of B solvent. Within 10 min, a linear gradient to 5% A / 95% B was programmed, and 5% A / 95% B was maintained for 1 min. Subsequently, a composition of 95% A / 5.0 % B was adjusted within 0.1 min and maintained for 2.9 min. The column oven was set to 40 °C, and the injection volume was 4 μl. The effluent was alternatively connected to an ESI-triple quadrupole-linear ion trap (QTRAP)-MS. LIT and triple quadrupole (QQQ) scans were acquired on a triple quadrupole-linear ion trap mass spectrometer (Q TRAP), API 4500 Q TRAP LC/MS/MS System, equipped with an ESI Turbo Ion-Spray interface, operating in positive and negative ion mode and controlled by Analyst 1.6.3 software (AB Sciex). The ESI source operation parameters were as follows: ion source, turbo spray; source temperature 550 °C; ion spray voltage (IS) 5500 V (positive ion mode)/-4500 V (negative ion mode); ion source gas I, gas II, and curtain gas were set at 50, 60, and 30.0 psi, respectively. The collision gas was high. Instrument tuning and mass calibration were performed with 10 and 100 μmol/L polypropylene glycol solutions in QQQ and LIT modes, respectively. QQQ scans were acquired as MRM experiments with collision gas (nitrogen) set to 5 psi. DP and CE for individual MRM transitions was done with further DP and CE optimization. A specific set of MRM transitions were monitored for each period according to the metabolites eluted within this period. Feature extraction and pre-processing of the raw data were conducted using XCMS^33^, and then normalized and edited into a two-dimensional data matrix in Excel 2010. Retention index (RT), mass-to-charge ratio (MZ), observations (samples) and peak intensity were calculated. Multivariate analysis (PCA) was performed using SIMCA-P 13.0 software (Umetrics AB, Umea, Sweden). Metabolites were assigned to pathways using the KEGG database. Significantly different metabolic pathways were identified using the PLS-DA (Partial Least Squares Discrimination Analysis), and the following criteria: VIP (Variable Importance in the Projection) value > 1 and P-value < 0.05. Data were processed and analysed by the Wuhan Metware Biotechnology Co., Ltd. (Wuhan, China).

### Statistical analyses

All values are presented as mean ± SD. The significance of differences between means was determined in Excel using Student’s *t-*test, with *P* < *0.05* as the threshold.

## Results

We collected fruits of *L. barbarum* and *L. ruthenicum* at five developmental stages, from young fruit (≈10 days post-flowering) to mature (ripe) fruit (34–45 days post-flowering), and studied their transcriptome and metabolome.

### RNA-seq *de novo* assembly and functional annotation of unigenes

**Figure 2:**
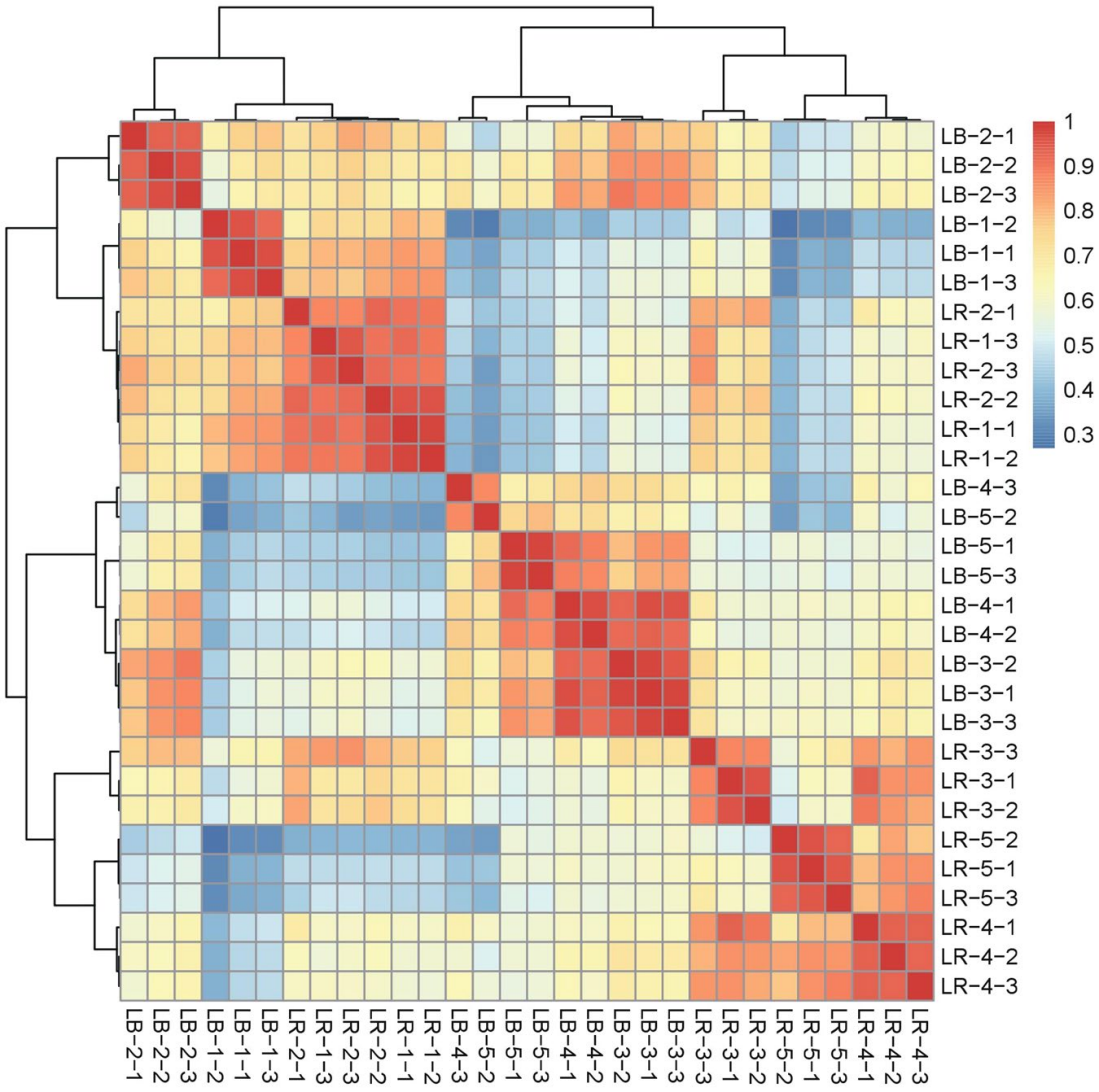
Heatmap of correlation coefficients for RNA-seq data for 30 samples of *L. barbarum* (LB) and *L. ruthenicum* (LR) fruits at five different developmental stages. Samples are labelled LB/R1–5_1–3, where LB is *L. barbarum*, LR is *L. ruthenicum*, 1–5 are fruit developmental stages, and 1–3 individual samples. Samples were grouped by hierarchical clustering; dendrograms above and left of the heatmap indicate relatedness of samples.

We prepared a total of 30 cDNA libraries from fruits of *L. barbarum* and *L. ruthenicum*, with three biological replicates (three fruits from three trees) at each time point: 2 species × 5 time-points × 3 biological replicates. Samples were labelled LB/LR(1–5)-(1–3), where LB is *L. barbarum* and LR is *L. ruthenicum*, 1–5 are developmental stages of fruit (S1–S5), and 1–3 are individual samples (biological replicates); so for example LB1-1 represents *L. barbarum*, 1^st^ sampled developmental stage (S1), fruit sample No.1 (out of three). We generated over 1.72 billion pair-end reads for these 30 cDNA libraries, corresponding to an average of 57.2 million reads per sample (Supplementary Dataset S1). Stringent quality assessment and data filtering yielded a total of 801,766 high-quality reads with the average length of 730 and N50 of 1107 bp (Table 1). Finally a total of 326,276 unigenes with the average length of 596 bp and N50 of 847 bp were obtained from the transcripts (Table 1). Correlation coefficients for RNA-seq data for the 30 samples indicate very good consistency of results among biological replicates (Fig. 2).

**Table 1:** Characteristics of assembled transcripts and unigenes.

Length (bp)	Transcripts	Unigenes
200–500	437,035	213,009
500–1 kbp	189,544	65,557
1 k–2 kbp	124,529	34,372
>2 kbp	50,658	13,338
Total	801,766	326,276
N50	1,107	847
Average	730	596
Min	201	201
Median	450	354
Max	17,104	17,104
Total nucleotides	585,430,380	194,467,186

Among all 326,276 unigenes queried against public databases, a total of 193,021 (59.15%) matched genes and/or proteins in at least one database, and 12,171 (3.73%) were annotated in all databases. The largest number of unigenes (149,863, 45.93%) was annotated in the NT database, and the lowest number (24,017; 7.36%) in the KOG database.

### *Lycium barbarum* (LB): pairwise interstage analyses

#### LB Transcriptome

**Figure 3:**
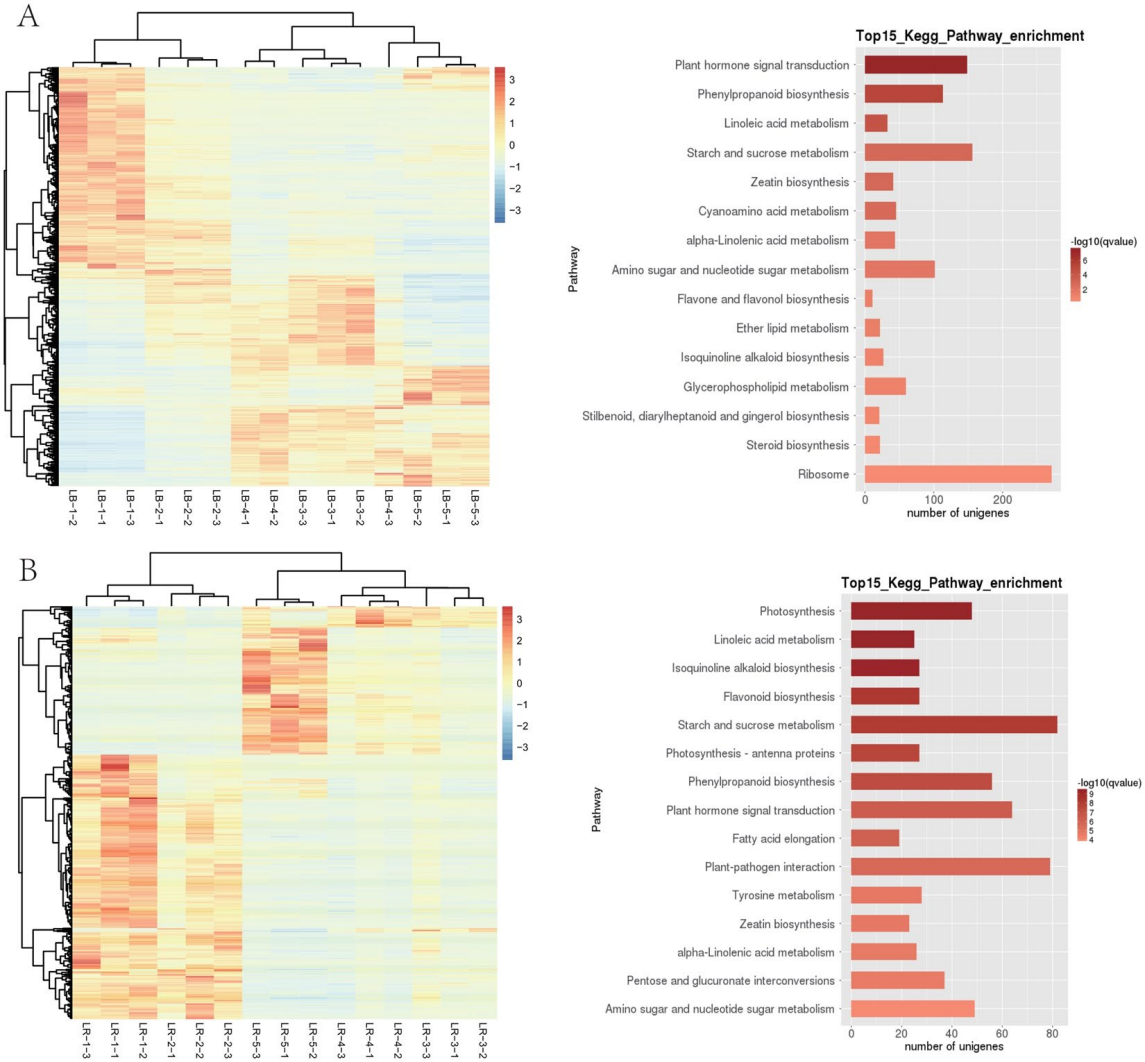
Heatmaps and functional pathway analyses of differentially expressed genes (DEGs) in *Lycium barbarum* (panel A) and *L. ruthenicum* (panel B) fruits. Heatmaps were generated by a hierarchical analysis of DEGs (y-axis) and individual samples (x-axis), where dendrograms above and left of the heatmap indicate relatedness of samples. Samples are labelled LB/R_1–5_1–3, where the species acronym (LB or LR) is followed by the developmental stage of fruit (1–5), and the sample number (1–3). Intraspecific KEGG pathway analyses of DEGs in all five developmental stages in the two species are shown to the right of the heatmaps. Only the top 15 enriched pathways are listed. q-value is an FDR-adjusted p-value.

The highest numbers of DEGs were identified in all pairwise comparisons of the 1^st^ stage, and in the 2^nd^ vs. 5^th^ stage comparison (all > 10,000 DEGs; Table 2). The smallest numbers were identified in 3^rd^ vs. 4^th^ and 4^th^ vs. 5^th^ stage comparisons (255–257). Heatmap analysis of DEGs in LB shows that fairly different sets of genes were highly upregulated in the early developmental stages (1 + 2) and in later stages (3 to 5) (Fig. 3A). Sample relatedness analysis indicates that the samples could be divided into two clades (stages 1 + 2 and 3 + 4 + 5), with the latter clade further subdivided into two clades: stages 3 + 4 and stage 5. Intraspecific KEGG functional classification analysis of these DEGs identified 15 pathways significantly (P < 0.05) differentially regulated among different developmental stages (Supplementary Dataset S2). Particularly strongly differentially regulated were ‘plant hormone signal transduction’, ‘phenylpropanoid biosynthesis (b.)’, ‘linoleic acid metabolism (m.)’, ‘starch and sucrose m.’, and ‘zeatin b.’ (Fig. 3A).

**Table 2:** Total numbers of significantly regulated genes and metabolites in pairwise comparisons of developmental stages in the two studied species.

Stages	Transcriptome						Metabolome					
	*Lycium barbarum*			*Lycium ruthenicum*			*Lycium barbarum*			*Lycium ruthenicum*		
	All	Down	Up	All	Down	Up	All	Down	Up	All	Down	Up
1 vs 2	11327	5809	5518	966	159	807	117	35	82	117	36	81
1 vs 3	19590	9273	10317	7743	2979	4764	117	42	75	117	44	73
1 vs 4	13565	5293	8272	7047	2922	4125	117	38	79	119	39	80
1 vs 5	16066	6209	9857	9328	2970	6358	97	47	50	98	48	50
2 vs 3	8046	3787	4259	2574	1383	1191	100	57	43	102	59	43
2 vs 4	5528	1953	3575	2899	1703	1196	108	56	52	114	61	53
2 vs 5	11193	4406	6787	5973	2233	3740	129	87	42	133	90	43
3 vs 4	257	23	234	39	11	28	66	28	38	66	27	39
3 vs 5	5284	1611	3673	3111	1073	2038	102	66	36	105	67	38
4 vs 5	255	114	141	783	103	680	104	72	32	104	71	33

**Figure 4:**
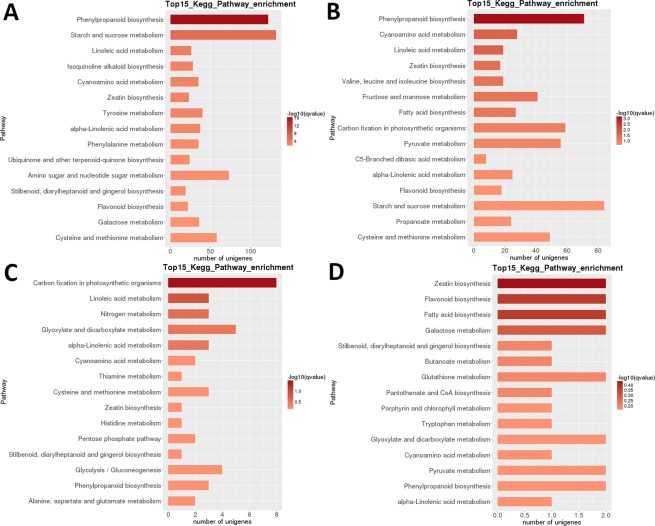
LB transcriptome: pairwise interstage KEGG metabolic pathway enrichment. (**A**) Stage 1 vs. stage 2. (**B**) Stage 2 vs. stage 3. (**C**) Stage 3 vs. stage 4. (**D**) Stage 4 vs. stage 5. Top 15 (or all if total <15) significantly enriched pathways are shown. Colour of the bar represents the magnitude of the q-value (an FDR-adjusted p-value). Colour chart is shown in the figure.

For a more in-depth analysis of the data, we focused on the comparison of most significantly regulated pathways in successive developmental stages. In the first pairwise comparison (S1 vs S2), ‘phenylpropanoid b.’ was the most highly differentially regulated pathway, followed by ‘starch and sucrose m.’ (Fig. 4). Very large numbers of DEGs (>100) were identified in both pathways. A similar result was observed in the following pairwise comparison, S2 vs. S3, but despite the fairly large number of DEGs (>80) ‘starch and sucrose m.’ exhibited somewhat lower q-value. In the In the S3 vs. S4 comparison, ‘carbon fixation in photosynthetic, organisms’ was the most significantly regulated pathway, but the numbers of genes were much lower. In the last pair, S4 vs. S5, ‘zeatin b.’, flavonoid b.’, fatty acid b.’ and ‘galactose m.’ were the most significantly regulated pathways, but none of the pathways exhibited more than two DEGs.

#### LB Metabolome

**Figure 5:**
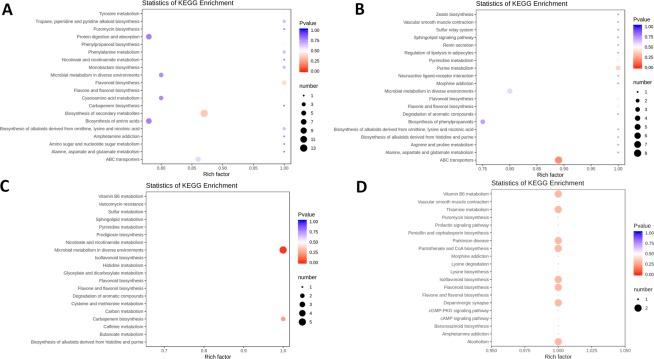
LB metabolomic data: pairwise interstage KEGG metabolic pathway enrichment. (**A**) Stage 1 vs. stage 2. (**B**) Stage 2 vs. stage 3. (**C**) Stage 3 vs. stage 4. (**D**) Stage 4 vs. stage 5. Rich factor is the ratio of the number of significantly regulated metabolites in the pathway and the total number of metabolites annotated in that pathway (range = 0 to 1.0). The size of the point represents the number of significantly enriched metabolites in the corresponding pathway, and the colour of the point represents the P-value (both legends shown in the figure).

We conducted pairwise developmental stage comparisons to identify the enrichment of metabolites between all stage pairs. The number of differentially regulated metabolites in pairwise comparisons of developmental stages in LB varied from 66 (stages 3 vs 4) to 129 (stages 2 vs 5). The largest number in successive stage comparisons was observed between 1^st^ and 2^nd^ stage (117). These were assigned to a large number of pathways; with the largest number of metabolites assigned to ‘b. of secondary metabolites’ (also the lowest p-value), followed by ‘protein digestion and absorption’ and ‘b. of amino acids’ (Fig. 5; Supplementary Figures). In the successive stage pair comparison (S2 vs. S3), ‘ABC transporters’ was the most significantly enriched pathway, followed by ‘purine m.’. In the S3 vs. S4 pair comparison, ‘microbial m. in diverse environments’ was the most significantly enriched pathway, followed by ‘carbapenem m.’. In the S4 vs. S5 pair comparison, a relatively large number of pathways exhibited similar results (two metabolites and similar p-values), but notable is the appearance of ‘isoflavonoid b.’ and ‘flavonoid b.’ among them (Fig. 5; Supplementary Figures).

As regards individual metabolites, in the S1 vs. S2 comparison, the list of metabolites upregulated in the S1 was topped by Trehalose, Glactinol and L-Malic acid (all ≈20 log2FC). Oleic acid, 2-Oxoadipic acid and Stearic acid were the most highly upregulated metabolites in S2 (all ≈15 to 18 log2FC). The list of most highly upregulated metabolites in S2 compared to S3 was topped by Dihydroxyacetone, LysoPC(18:1(9Z)), and Adenine (all ≈16 to 17.5 log2FC). The list of most highly upregulated metabolites in the S3 (compared to S2) was topped by Trehalose, Galactinol and L-Malic acid (all ≈19 to 20 log2FC). In the S3 vs. S4 comparison, the list of most highly upregulated metabolites in the S3 was topped by L-Malic acid, DL-Arginine and Oleic acid (all ≈18 to 19 log2FC), whereas the list of most highly upregulated metabolites in the S4 was topped by D-Mannose, N-Acetyllactosamine and LysoPC(18:1(9Z)) in LB (all ≈16 log2FC). In the S4 vs. S5 comparison, the list of most highly upregulated metabolites in the S4 was topped by Trehalose, Palmitic acid, N-Acetyllactosamine (all ≈17 to 18 log2FC) in LB. The list of most highly upregulated metabolites in the S5 was topped by L-Norleucine, Anthranilic acid (Vitamin L1) and DL-Arginine (all ≈16 to 18 log2FC) (Supplementary Dataset S3).

### *Lycium ruthenicum* (LR): pairwise interstage analyses

#### LR Transcriptome

**Figure 6:**
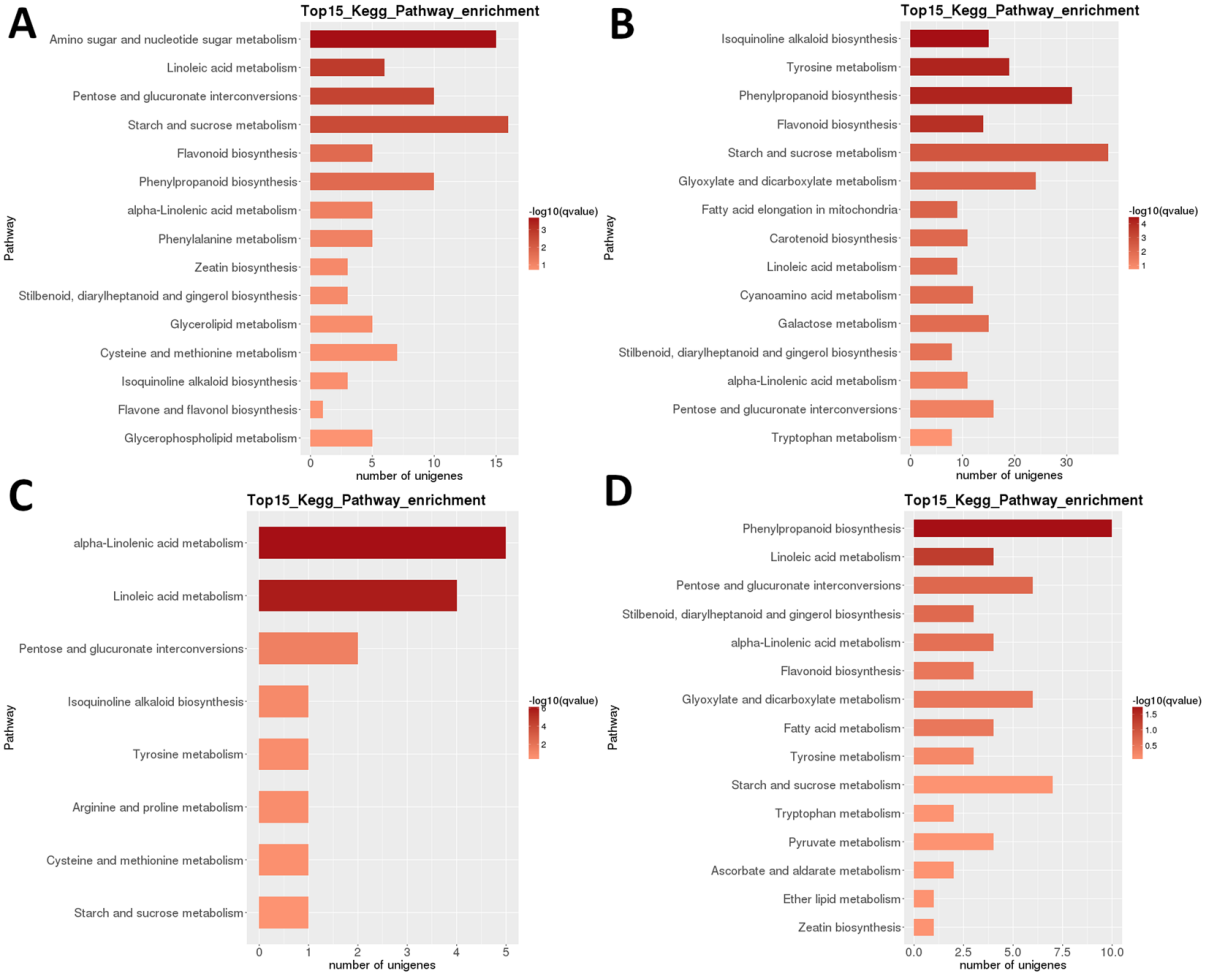
LR transcriptome: pairwise interstage KEGG metabolic pathway enrichment. (**A**) Stage 1 vs. stage 2. (**B**) Stage 2 vs. stage 3. (**C**) Stage 3 vs. stage 4. (**D**) Stage 4 vs. stage 5. Top 15 (or all if total <15) significantly enriched pathways are shown. Colour of the bar represents the magnitude of the q-value (an FDR-adjusted p-value, colour chart shown in the figure).

Heatmap analysis of DEGs in LR shows that almost completely different sets of genes were highly upregulated in the early developmental stages (1 + 2) and in ripe fruit (stage 5), with an apparent transcriptomic reset occurring after the second stage (Fig. 3B). Sample relatedness analysis indicates that the samples could be divided into two clades (stages 1 + 2 and 3 + 4 + 5), with the latter clade further subdivided into two clades: stages 3 + 4 and stage 5. Intraspecific KEGG functional classification analysis of these DEGs identified 35 pathways significantly (P < 0.05) differentially regulated among different developmental stages (Supplementary Dataset S2). Particularly strongly differentially regulated were ‘photosynthesis’, ‘linoleic acid m.’, ‘isoquinoline alkaloid b.’, ‘flavonoid b.’ and ‘starch and sucrose m’ (Fig. 3B). The highest numbers of DEGs were identified in most pairwise comparisons of the stage 1 (1 vs. 2 was an exception), and in the stage 2 vs. 5 comparison (all >5,000 DEGs; Table 2). By far the smallest number was identified in the S3 vs. S4 comparison (39). In the S1 vs S2 pairwise comparison, ‘amino sugar and nucleotide sugar m.’ was the most highly differentially regulated pathway, followed by ‘linoleic acid m.’ (Fig. 6). In the S2 vs. S3 pairwise comparison, the most significantly differentially regulated pathways were ‘isoquinoline alkaloid b.’, ‘tyrosine m.’, ‘phenylpropanoid b’, and ‘flavonoid b.’. In both pairwise comparisons, the highest number of DEGs (>15 and >35 respectively) was identified in the ‘starch and sucrose m.’. In the S3 vs. S4 comparison, ‘(alpha-)linoleic acid m.’ was the most significantly regulated pathway, but the numbers of genes were much lower. In the S4 vs. S5 comparison, ‘phenylpropanoid b’ (also the largest number of DEGs), and ‘linoleic acid m.’ were the most significantly regulated pathways.

#### LR Metabolome

**Figure 7:**
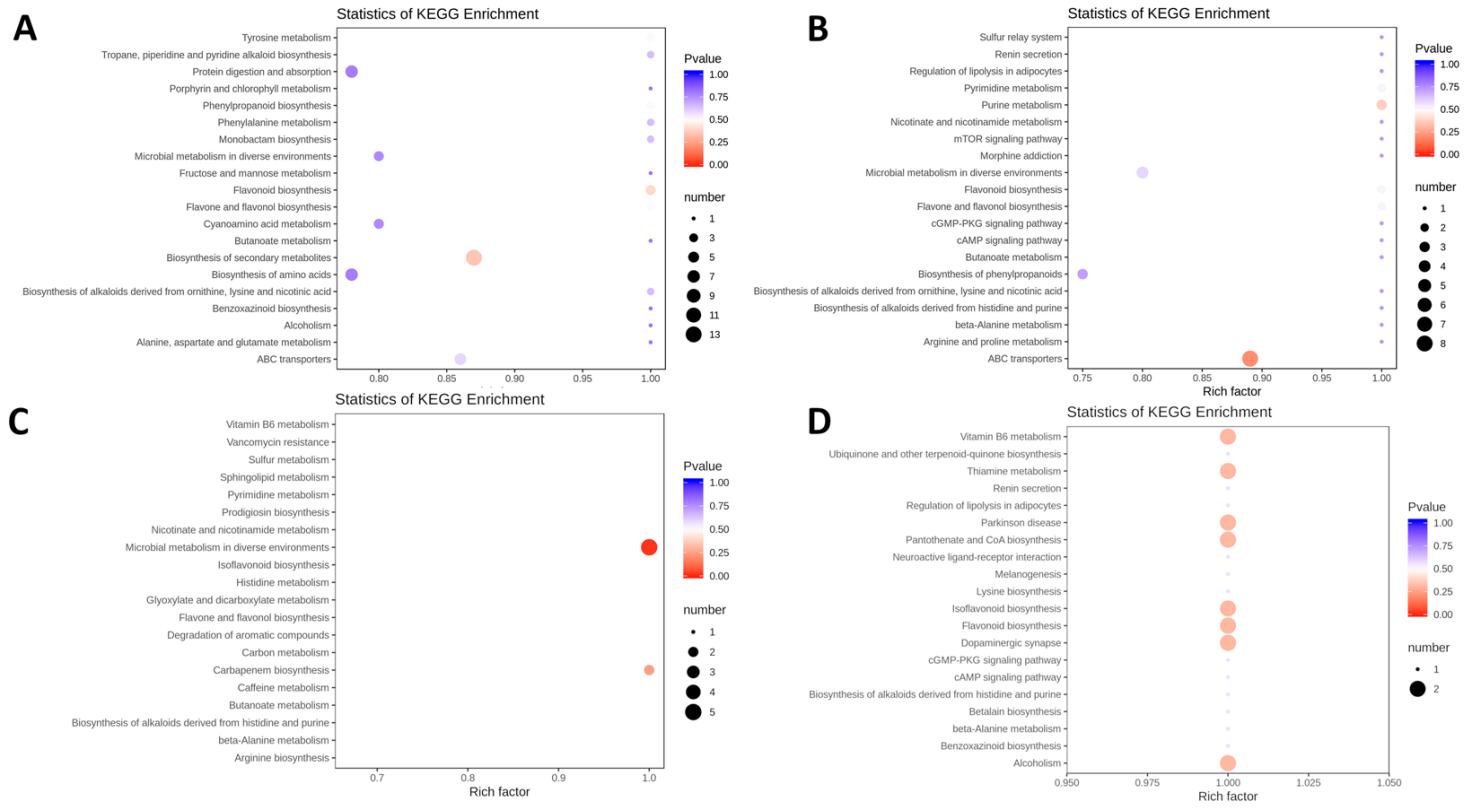
LR metabolomic data: pairwise interstage KEGG metabolic pathway enrichment. (**A**) Stage 1 vs. stage 2. (**B**) Stage 2 vs. stage 3. (**C**) Stage 3 vs. stage 4. (**D**) Stage 4 vs. stage 5. Rich factor is the ratio of the number of significantly regulated metabolites in the pathway and the total number of metabolites annotated in that pathway (range = 0 to 1.0). The size of the point represents the number of significantly enriched metabolites in the corresponding pathway, and the colour of the point represents the P-value (both legends shown in the figure).

The number of differentially regulated metabolites in pairwise comparisons of developmental stages in LR varied from 66 (stages 3 vs 4) to 133 (stages 2 vs 5) (Supplementary Dataset S3). In successive stage comparisons, the largest number was observed between 1^st^ and 2^nd^ stage (117). These were assigned to a large number of pathways; with the largest number of metabolites assigned to ‘b of secondary metabolites’, followed by ‘protein digestion and absorption’, ‘b. of amino acids’, and ‘flavonoid b’. In the S2 vs. S3 comparison, ‘ABC transporters’ was the most significantly enriched pathway, followed by ‘purine m’. In the S3 vs. S4 pair comparison, ‘microbial m. in diverse environments’ was the most significantly enriched pathway, followed by ‘carbapenem m.’. In the S4 vs. S5 pair comparison, a relatively large number of pathways exhibited similar results (2 metabolites and similar p-values), but notable is the appearance of ‘isoflavonoid b.’ and ‘flavonoid b.’ among them (Fig. 7; Supplementary Figures).

As regards individual metabolites (Supplementary dataset S3), in the S1 vs. S2 comparison, the list of metabolites upregulated in the S1 was topped by Trehalose, Galactinol and L-Malic acid (≈19–21 log2FC), whereas Oleic acid, 2-Oxoadipic acid and Stearic acid were the most highly upregulated metabolites in S2 (≈15–18 log2FC). In the S2 vs. S3 comparison, Dihydroxyacetone, Indoxyl sulphate and N-Acetyllactosamine were most highly upregulated metabolites in the S2 (≈17–19.5 log2FC), and Trehalose, Galactinol and L-Malic acid (≈19–21 log2FC) in the S3. In the S3 vs. S4 comparison, L-Malic acid, DL-Arginine and Oleic acid were upregulated in the S3 (≈16–19 log2FC), and 1,7-Dimethylxanthine, D-Mannose and N-Acetyllactosamine (≈15–17 log2FC) in the S4. In the S4 vs. S5 comparison, Flavin mononucleotide, Trehalose and Isoferulic acid were upregulated in the S4 (≈18–20 log2FC), whereas PG(16:0/18:1(9Z)), D-Proline, and DL-Arginine were most highly upregulated (all ≈16 to 18 log2FC) metabolites.

### Interspecific comparative analysis of DEGs at different developmental stages

#### Total DEGs during the fruit development

**Figure 8:**
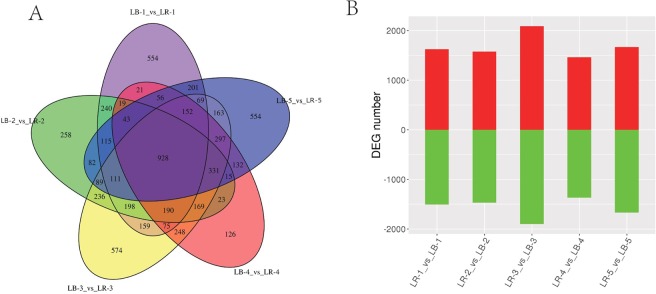
Genes differentially expressed (DEGs) between fruits of *L. barbarum* (LB) and *L. ruthenicum* (LR). (**A**) Detailed stagewise (1 to 5) comparisons (LB vs. LR). (**B**) The number of upregulated (red) and downregulated (green) DEGs in LR in comparison to LB in five studied developmental stages.

Interspecific pairwise stage comparison (LR1 vs. LB1, LR2 vs.LB2, etc.) shows that 928 DEGs were shared by all five pairs (Fig. 8A). The highest number of DEGs was identified in stage 3 (3989), and the lowest in stage 4 (2825) (Fig. 8B); whereas the highest numbers of DEGs unique to a pair were observed in stages 3 (574), 1 and 5 (both 554), and the lowest in stage 4 (126) (Fig. 8A). The numbers of up- and down-regulated DEGs were relatively similar in each of the pairwise stage comparisons; e.g., in stage 5, 1668 DEGs were upregulated and 1670 DEGs were downregulated in LR in comparison to LB (Fig. 8B). However, in the other four stages the number of upregulated genes was slightly (93 to 189 DEGs) higher.

#### Transcriptome – pathways

**Figure 9:**
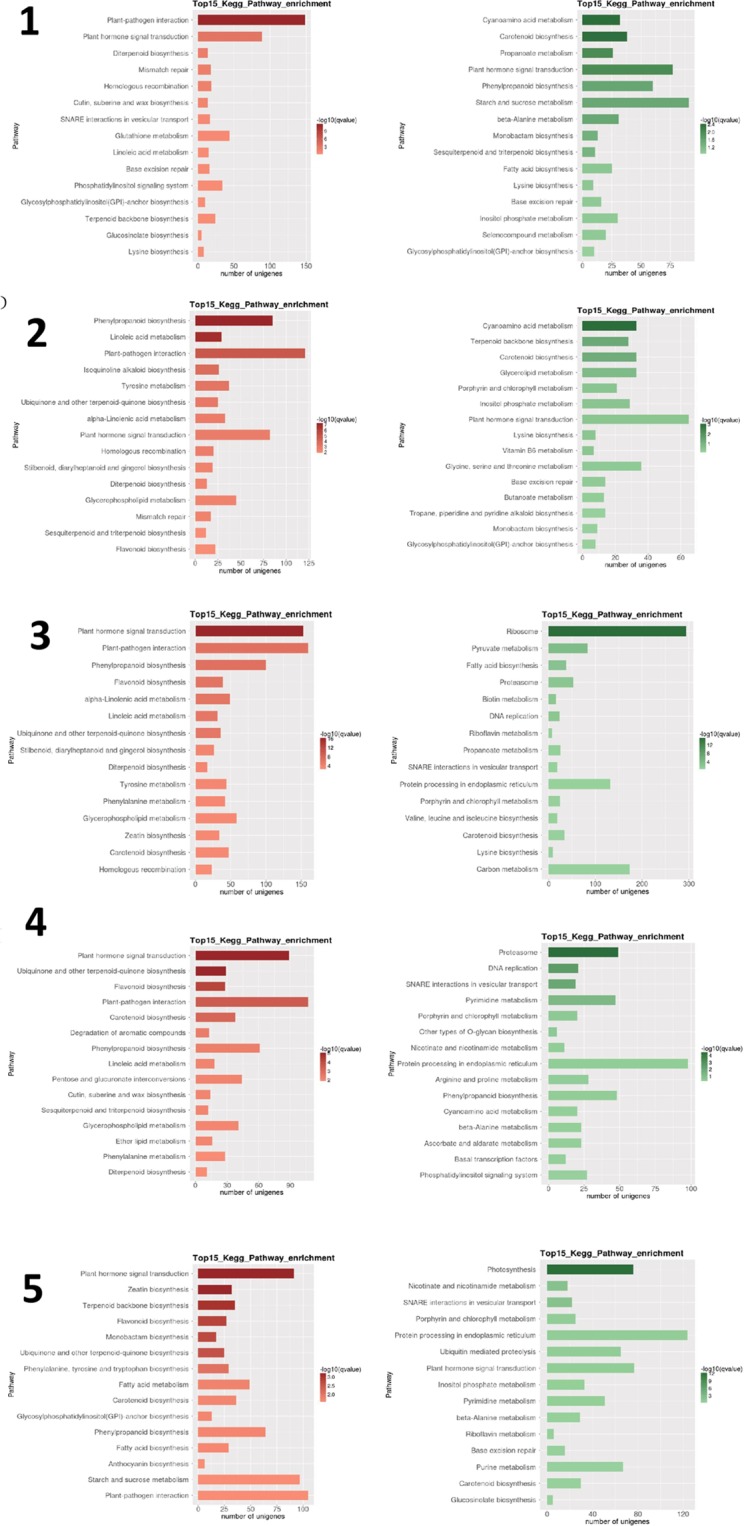
Comparative analysis of KEGG metabolic pathway enrichment. The top 15 pathways enriched in *L. ruthenicum* in comparison to *L. barbarum* are shown on the left (red), and the ones enriched in *L. barbarum* in comparison to *L. ruthenicum* on the right (green). Developmental stages (1–5) are indicated in the figure. q-value is an FDR-adjusted p-value.

Heatmap analysis of DEGs indicates that the fruits of two species exhibit very different gene expression profiles during all developmental stages, but biological replicates exhibited very similar profiles, indicating a limited amount of individual variability in each developmental stage (Supplementary Figures: Fig. S9). Comparative analysis of KEGG pathway enrichment shows that only some pathways were consistently highly enriched (in terms of gene regulation) in LR in comparison to LB throughout all five developmental stages (Fig. 9). Notably, plant hormone signal transduction (2^nd^-highest in S1, 8^th^-highest in S2, the highest in S3, S4 and S5) and plant-pathogen interaction (the highest in S1, 3^rd^-highest in S2, 2^nd^-highest in S3, 4^th^-highest in S4, and 15^th^-highest in S5) were relatively highly upregulated in all stages. Phenylpropanoid biosynthesis (not in top 15 in S1, the highest in S2, 3^rd^-highest in S3, 7^th^-highest in S4, 11^th^-highest in S5), ubiquinone and other terpenoid-quinone biosynthesis (not in top 15 in S1, 6^th^-highest in S2, 7^th^-highest in S3, 2^nd^-highest in S4, 6^th^-highest in S5) were also relatively highly upregulated in all stages except the first one. Flavonoid biosynthesis pathway was not highly enriched in early stages (not in top 15 in S1, 14^th^-highest in S2), and highly enriched in late stages (3^rd^ to 4^th^-highest during stages 3 to 5). (alpha-)Linoleic acid metabolism was highly enriched in middle stages (9^th^-highest in S1, 2^nd^-highest in S2, 5^th^ and 6^th^-highest in S3, 8^th^-highest in S4, not in top 15 in S5).

Among the pathways downregulated in *L. ruthenicum* in comparison to *L. barbarum* (Fig. 9) notable changes were observed between the early stages (1 and 2), when cyanoamino acid metabolism and carotenoid biosynthesis were most highly downregulated, and late stages (4 and 5), when SNARE interactions in vesicular transport, nicotinate and nicotinamide metabolism, and porphyrin and chlorophyll metabolism were consistently relatively highly downregulated.

#### Transcriptome – individual genes

Among the most highly differentially expressed genes, some were developmental stage-specific (i.e. highly differentially regulated only in early or in late developmental stages), but some were consistently highly differentially regulated throughout all five studied stages (Supplementary Dataset S4). Several immunity-related genes very highly upregulated in LR in comparison to LB in the early developmental stages are among the examples of the developmental stage-specific expression pattern: *chitinase* was the 2^nd^ highest upregulated DEG in S1 (13.43-fold), the highest in S2 (13.89-fold), but in later stages it was not a DEG. Similarly, *EIX receptor 1/2* was also very highly upregulated in early stages, the highest in S1 (13.70) and 2^nd^-highest in S2 (10.90), but it was also not identified as DEG in later stages. Some growth-related genes also exhibited a similar expression pattern: *phosphoglycerate kinase* (*PGK*) was among the handful of most highly upregulated genes in the first three stages (13.14, 12.87 and 12.77 respectively), but it was also not identified as DEG in later stages. *CCR4-NOT transcription complex subunit 7/*8 (*CNOT7*/*8*) also exhibited a very similar expression pattern: highly upregulated in first three stages, and not a DEG in stages 4 and 5. Several flavonoid and phenylpropanoid biosynthesis-associated genes exhibited a reversed developmental stage-specific expression pattern, with relatively low expression in early stages, and very high in later stages. Examples are: *bifunctional dihydroflavonol 4-reductase/flavanone 4-reductase* (*DFR*), which was slightly upregulated in LR in S1 (2.25), not a DEG in S2, highly upregulated in S3 (7.79), and 3^rd^-highest upregulated gene in S4 (14.25) and S5 (16.03). A paralogue of this gene exhibited an almost identical pattern: slightly upregulated in S1 (2.44), not a DEG in S2, highly upregulated in S3 (7.40), 6^th^-highest upregulated DEG in S4 (13.26) and 5^th^ in S5 (14.59). Similarly, *flavonoid 3*′*,5*′*-hydroxylase* (*F3*′*5*′*H*) was not a DEG in the first two stages, highly upregulated in S3 (6.69), 5^th^-highest upregulated gene in S4 (13.42), and 4^th^-highest in S5 (15.05). *Flavonoid O-methyltransferase* (*OMT*) was not a DEG in the S1, but in S2 it already exhibited a medium-high upregulation level (4.32), by the S3 it was already the third-highest upregulated DEG (13.30), and it was the highest-upregulated gene in S4 (18.73) and S5 (18.10). *Leucoanthocyanidin dioxygenase* (*LDOX*; anthocyanin biosynthesis) was not a DEG in S1 and S2, followed by high to very high upregulation in later stages (5.63, 9.44, 11.56, respectively). Two *chalcone synthase* paralogues (*CHS* and *CHS2*; flavonoid biosynthesis) were also not highly regulated in S1 and S2 (*CHS2*: not a DEG, *CHS*: −1.14 in S1, not a DEG in S2), but in S3–S5 both genes exhibited a medium-high to high upregulation (*CHS2*: 5.32, 7.84, 6.00; and *CHS*: 4.67, 7.01, 6.82; respectively). We selected these genes for qPCR analysis, and the results are highly congruent with the RNA-seq data (Supplementary Results; Supplementary Dataset S5). Finally, *cytokinin dehydrogenase*, a zeatin biosynthesis-related gene, was also increasingly upregulated during the last three stages (2.6–5.7).

However, some genes were consistently differentially expressed throughout all five studied stages. Examples also included some immunity-related genes, such as two paralogues of *glutathione S-transferase*, highly upregulated in LR in comparison to LB in all stages: 9.38 and 8.58 (all values presented as fold-changes in respective order) in S1, 6.30 and 6.34 in S2, the 2^nd^ and 7^th^ highest upregulated DEGs in S3 (14.08 and 12.70), 2^nd^ and 4^th^ highest in S4 (15.71 and 14.16) and 2^nd^ and 6^th^ highest in S5 (16.48 and 14.40). *Plant disease resistance protein RPM1* was also highly upregulated in all five stages (S1 = 13.15; S2 = 12.08; S3 = 13.11, S4 = 12.81; S5 = 13:94). Among the consistently differentially expressed genes throughout all developmental stages were also some related to the amino acid metabolism, but their pattern was reversed in comparison to previous examples: they exhibited high downregulation in LR compared to LB. Examples are *acetyl-CoA acyltransferase 1* (*AAT1*; valine, leucine and isoleucine degradation), with a temporal profile of increasingly high downregulation, starting from −7.0 in the S1 to <−10-fold in the last three stages. *Proline iminopeptidase*, associated with arginine and proline metabolism, was highly downregulated in LR in all stages: S1 = −9.75, S2 = −10.89 (3^rd^-highest), S3 = −11.05 (4^th^-highest), S4 = −10.01, and S5 = −11.98 (3^rd^-highest). Finally, *5-methyltetrahydropteroyltriglutamate–homocysteine methyltransferase* (*metE*) was consistently extremely highly downregulated in LR in all stages: 2^nd^-highest in S1 (−11.76), the highest in S2 (−11.74), 3^rd^-highest in S3 (−11.43), the highest in S4 (−12.36), and 2^nd^-highest in S5 (−12.83). Two DNA replication and transcription-associated genes were also highly downregulated in LR in all stages: *GTP-binding nuclear protein Ran* (*RAN*; −10.0 to −12.0) and *replication factor A1* (*RFA1*) (−8.0 to −12.0). Some growth and stress-related genes were also consistently highly downregulated in LR: *heterogeneous nuclear ribonucleoprotein A1/A3* (*hnRNP*; −7 to −11) and *heat shock 70 kDa protein 1/8* (*HSPA1_8*) S1 = −4.95, S2 = −8.88, S3 = −11.48 (2^nd^-highest), S4 = −9.36, S5 = −12.89 (the highest). Intriguingly, a phenylpropanoid biosynthesis-related gene, *shikimate hydroxycinnamoyltransferase* (*HCT*), was also consistently highly downregulated in LR: S1 = −6.82, S2 = −8.14, S3 = −11.71 (the highest), S4 = −11.00 (3^rd^-highest), S5 = −11.91 (4^th^-highest). However, a key regulator of anthocyanin biosynthesis, transcription factor MYB114, was highly upregulated in LR during all five developmental stages: 6.11, 4.69, 7.47, 9.05, and 8.95 (S1–S5 respectively).

#### Metabolome – pathways

**Figure 10:**
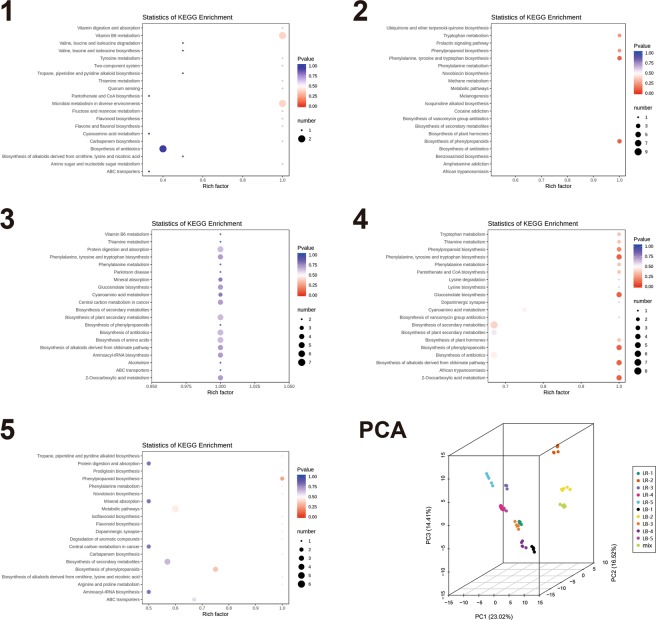
Comparative analysis of KEGG metabolic pathway enrichment. The top 15 pathways enriched in *L. ruthenicum* in comparison to *L. barbarum* are shown on the left (red), and the ones enriched in *L. barbarum* in comparison to *L. ruthenicum* on the right (green). Developmental stages (1–5) are indicated in the figure. q-value is an FDR-adjusted p-value.

We also conducted a comparative interspecific stage-wise analysis of metabolic pathways (Fig. 10). In the first developmental stage (S1), we identified 39 differentially regulated metabolites. Among the top 20 pathways these metabolites were associated with, several of them were associated with amino acids, but total numbers of metabolites per pathway were relatively small (1–2), and P-values did not suggest a high level of significance (Fig. 10 - panel 1). Notably vitamin B6 metabolism (m) and microbial m in diverse environments both exhibited comparatively high P-values, enrichment factor (EF) of 1.0, and 2 identified metabolites. In the S2, we identified 58 differentially regulated metabolites, associated with only four pathways: tryptophan m, phenylpropanoid biosynthesis (b), b of phenylpropanoids (these are two different pathways in the KEGG database), and phenylalanine, tyrosine and tryptophan b (all EF = 1.0, 2–3 metabolites, and p < 0.5; Fig. 10 - panel 2). In the S3, we identified 59 differentially regulated metabolites, associated with 19 pathways, most all with the EF 1.0, but relatively non-significant P-values (>0.5; Fig. 10 - panel 3). Pathways with relatively high number of metabolites (n = 5) were: protein digestion and absorption, b of plant secondary metabolites, b of antibiotics, and b of amino acids. In the S4, we identified 58 differentially regulated metabolites, associated with a large number of pathways, mostly with the EF 1.0, and comparatively high significance values (mostly P > 0.5; Fig. 10 - panel 4). Pathways with relatively high number of metabolites (n ≥ 3) were: phenylpropanoid b, phenylalanine, tyrosine and tryptophan b, glucosinate b, b of alkaloids derived from shikimate pathway, and 2-oxocarboxylic m. In the ripe fruit (S5), we identified 39 differentially regulated metabolites, associated with a large number of pathways, but mostly with low P-values and only 1 metabolite per pathway (Fig. 10 - panel 5). Pathways with more than 1 metabolite were: protein digestion and absorption, phenylpropanoid b, mineral absorption, central carbon m in cancer, b of secondary metabolites, b of phenylpropanoids, and aminoacy tRNA b. The principal component analysis (PCA) of all data (2 species × 5 stages × 5 biological replicates) revealed high similarity among biological replicates (clustering), and corroborated notable variability between different fruit ripening stages for both species (Fig. 10 – panel 6).

#### Metabolome - individual metabolites

The list (Supplementary Dataset S6) of most highly differentially regulated metabolites between the two species, exhibited some variation among the five fruit development stages. Intriguingly, Fructose 1-phosphate was the most highly upregulated metabolite in LR, compared to LB, during all five stages: log2 Fold change = 6.3, 7.6, 7.7, 8.1, and 6.5 (stages 1 to 5 respectively). As regards the metabolites highly upregulated in LB, there was more variation among stages: in the S1, differences were rather small, with 9-Decenol as the most highly upregulated metabolite (log2 Fold change = 2.7; compared to LR). Analyses of the S2 and S3 produced highly congruent results, with phenol (3.7 and 3.2 respectively) as the most highly upregulated metabolite. In the S4, indoxyl sulphate (4.7) was the top metabolite upregulated in LB. A metabolic shift was observed in the ripe fruit (S5), where the list of metabolites upregulated in LB was topped by by stearoylcarnitine (7.1), Methoxyacetic acid (5.3), S-Methyl-5′-thioadenosine (4.7), lisinopril (4.7), Adenosine 3′,5′-cyclic phosphate (cAMP) (4.7), etc. Other metabolites highly upregulated in LR (apart from Fructose 1-phosphate) were naringin (6.2), lauroyl-CoA (4.8), L-Phneylalanine (4.6), etc.

## Discussion

### Transcriptome and metabolome assembly quality

Given the absence of a published genome for this species, almost 60% of unigenes matching against genes and/or proteins in at least one database is a satisfactory annotation rate. For example, a recent transcriptomic study of *L. barbarum*^6^ produced an almost identical number of genes annotated in all databases (12,246) as our study (12,171). This similarity, high correlation coefficients among biological replicates, and additional confirmatory qPCR experiments, all corroborate that the transcriptome sequencing experiment and data analysis were conducted to a high standard. As gene expression (mRNA levels) can be variable over very short time-periods, RNA-seq often produces conflicting signals^34,35^, which is a likely explanation for some apparently simultaneously highly upregulated and highly downregulated pathways (such as carotenoid biosynthesis and plant hormone signal transduction in several stages) in the interspecific pairwise comparisons. This relative volatility in gene expression levels is also the most likely explanation for several observed instances of incongruence between the transcriptomic and metabolomic data; such as much higher variability in the successive intraspecific pairwise comparisons observed in the transcriptomic dataset.

A very large number of differentially regulated genes and metabolites belonging to a broad range of metabolic pathways reflects the fact that fruit ripening is a complex developmental process, characterized by a series of transitions that are coordinated by a network of interacting genes and signalling pathways^12^. Although we observed similar patterns in intraspecific pairwise comparisons of successive stages in the transcriptomic data, the total numbers of regulated DEGs were consistently smaller in LR, except for the 4^th^ vs. 5^th^ stage comparison, where a higher number of DEGs was identified (783). As this was not reflected in the metabolomic data, it is likely that this is an annotation artefact produced by a higher data availability for LB.

### Genes and metabolites that may be associated with abiotic stress responses

As LR is native to the salinized deserts of northwestern China, its genetics and physiology should bear strong markings of the evolution in an environment where drought and salt stress are very common. Indeed, it generally exhibits higher resistance to abiotic and biotic stressors common in that environment, such as high soil salinity, drought and local pests, than *L. barbarum*. A major abiotic stressor, drought, has received ample scientific attention, as it is rather common, and affects the productivity and growth of numerous economically important plants^36–38^. Although we are far from a full understanding of the complexities of these mechanisms, some genes/metabolites/metabolic pathways have been singled out as particularly highly affected by abiotic stress, examples of which are abscisic acid (ABA), hormone signal transduction, metabolisms of proteins, carbohydrates, nucleic acids and lipids, etc.^36–39^. Salt stress can indirectly impair photosynthesis by depressing chlorophyll biosynthesis and the citrate cycle, so *acetyl-CoA acyltransferase 1* (*AAT1*) is known to be strongly downregulated in response to salt stress^40^, which is in perfect agreement with very high downregulation in all stages observed in our results. A gene involved in the biosynthesis of amino acids, but also a number of important secondary metabolites in plants, *metE*, exhibited a temporal profile of increasingly high downregulation. Due to its varied metabolic roles there are multiple possible explanations for this, but as downregulation of this enzyme was observed in response to drought stress in other plants^41^, its regulatory pattern may also be related to the higher drought exposure of LR plants in our experimental setup.

In agreement with this observation are also the results of comparative interspecific individual metabolite analyses, where a number of metabolites associated with abiotic stress topped the list of most highly significantly differentially regulated metabolites between the ripe fruits of the two species (here we focus only on the ripe fruits, as they are more interesting than other studied stages from the human perspective). Plants counteract the deleterious effects of drought by accumulating osmolytes, such as amino acids, amines and some soluble carbohydrates (especially the raffinose family), which have a vital role for the stability of cellular structures under adverse environmental conditions^40,42,43^. Increased level of raffinose in post-colour breaking stages of fruit development in LR was observed recently, and associated with osmoregulation requirements^3^. In our study, raffinose was also increased in LR (3.6-fold), as was another raffinose family oligosaccharide, galactinol (11.7-fold). A heightened synthesis of galactinol has been reported in plants in response to a range of abiotic stressors, so it is believed to function as an osmoprotectant in drought-stress tolerance of plants^42–44^. Intriguingly, another carbohydrate highly enriched (8.09-fold) in LR, trehalose, is a disaccharide of glucose that functions as an osmoprotectant under abiotic stress in bacteria, fungi, and invertebrates, but it generally does not accumulate in detectable levels in most plants, with the exception of desiccation-tolerant “resurrection plants”^45,46^. Trehalose and galactinol also featured prominently in both species in the lists of most highly regulated metabolites in the intraspecific comparisons between different stages, suggesting a prominent role of these metabolites in both species. This is likely to be a reflection of a high level of adaptation to arid habitats in both species.

Among other examples of osmolytes abundant in LR are sucrose (37-fold) and betaine (28-fold). A member of the betaine family, glycine betaine, is an important organic osmolyte that protects cells against osmotic stress caused by drought or high soil salinity^47^. Sucrose is another highly significantly upregulated carbohydrate that (similar to trehalose) protects membranes and proteins in bacteria during drying^48^. A recent study found that sucrose was the most abundant sugar in both *Lycium* fruits before colour-breaking, but glucose and fructose were significantly elevated post colour-breaking in both fruits^3^. Therefore, the high levels of sucrose in ripe LR fruits observed in our study are likely to be a reflection of adaptation to drought and/or salinity stress.

Several metabolites related with amino acid metabolism- were also highly significantly more abundant in the ripe LR fruit: citrulline (31.1-fold), arginine (31.1-fold) and glutamate (29.5-fold). A massive accumulation of these three metabolites (especially citrulline) in response to a drought stress was observed in wild watermelon (*Citrullus lanatus*)^37,49^. The authors suggested that the accumulation of citrulline during the drought stress is a unique phenomenon in C_3_-plants. Although reports of the association between high citrulline accumulation and drought appear to be limited to watermelon species^37,49^, citrulline accumulation was associated with higher disease resistance in citrus fruits^50^. Glutamate and arginine are both precursors for citrulline synthesis^49^, and arginine is believed to play a role in the fine-tuning of stress defense mechanisms^51^. Arginine is also a precursor for nitric oxide and polyamines, which are important metabolites in stress responses^52^. For example, high arginine accumulation in response to long-term drought stress was also observed in chickpea^53^. Intriguingly, we observed a strong accumulation of DL-arginine in the ripe fruit of both species, but further studies are needed to assess whether this may be related to abiotic stress adaptation in both species.

Expression patterns of some genes were (apparently) not in agreement with the hypothesis of increased stress-related gene expression in LR. *Proline iminopeptidase* (*pip*), associated with arginine and proline metabolism, was highly downregulated in LR in all stages. As proline (and *pip*) concentrations tend to increase under stress in a broad range of living organisms^40,54,55^, we would expect the *pip* gene to be upregulated. It should be noted that we did observe a high accumulation of L-proline in the ripe LR fruit. However, a downregulation of *pip* in response to drought stress was also observed in *Bombax ceiba*^36^, and proline accumulation was triggered in *Calotropis procera* in response to salt stress, but not drought stress^56^, which indicates that this discrepancy may not be mere molecular noise, and might deserve further investigation.

### Genes and metabolites that may be associated with the accumulation of anthocyanins

LB and LR fruits are generally relatively rich in pharmacologically important secondary metabolites synthesized via the phenylpropanoid/flavonoid pathway, such as anthocyanin, betalain, flavone, flavonoid, isoquinoline, etc.^3,5,6,9^. Flavonoids have high antioxidant potential and possess a number of properties putatively beneficial from the pharmacological perspective: antitumorigenic, anti-inflammatory, prevention and treatment of cardiovascular and neurodegenerative diseases, obesity, dental health, etc.^5,57–60^. Phenylpropanoids, a group of phenylalanine-derived physiologically active secondary metabolites, are the key mediators of plant responses towards abiotic (such as light and soil minerals) and biotic (pests) stimuli, also with important (from the human health perspective) antioxidant and free radical scavenging properties^61^. Many functional pathways, genes and metabolites associated with phenylpropanoid and flavonoid biosynthesis exhibited upregulation in LR berries, especially in later developmental stages. Developmental stage-specific expression pattern, with relatively low expression in early stages, and very high in later stages was exhibited by the paralogues of *DFR* and *CHS*, as well as *F3*′*5*′*H*, *OMT* and *LDOX* genes, all of which take part in the biosynthesis of anthocyanin, natural pigment of plants, responsible for red, blue and purple colours^57,62,63^. These genes are commonly upregulated in later stages of fruit ripening^12,62^; for example, high expression of *DFR* increases the accumulation of anthocyanin content during fruit ripening^62,64^. Importantly, a key positive regulator of the anthocyanin biosynthesis, *transcription factor MYB114*^12,65^, was highly upregulated in LR during all five developmental stages. At the metabolomic level, a metabolite associated with flavone and flavonol biosynthesis pathway, rutin, was significantly more abundant in ripe LR fruits than in LB fruits (5.88-fold). Increased rutin content was observed in plants in response to a drought stress^66^, salinity stress^67^, and also to biotic stress (pests)^68^. Intriguingly, this metabolite was not mentioned in a recent comparative metabolomic study of these two *Lycium* fruits^3^. As rutin derivatives have antioxidant potential and show low cytotoxicity in human and animal cells, which makes them promising potential candidates for use as nutraceuticals^69^, and as there are indications that rutin may suppress lipid accumulation in humans^70^, high rutin content in LR berries may be interesting from the nutritional and pharmaceutical perspective.

## Conclusions

The interpretation of our findings is hampered by different environmental parameters at the two sampled locations, as well as by the fact that LB has undergone generations of anthropogenic selection for higher growth, whereas the genome of LR is likely to be shaped solely by non-anthropogenic factors. As it was difficult to disentangle genetic from environmental variables, and anthropogenic from non-anthropogenic variables in our study, we limited the discussion of our results to the most highly pronounced transcriptomic and metabolomic differences between the two species. Previous studies found indications that *L. ruthenicum* may exhibit higher resistance to abiotic (such as high soil salinity and drought) than *L. barbarum*^3,4,6,9,11^ and that berries of *L. ruthenicum* may have much greater medicinal value than berries of *L. barbarum*^2,3^. Although we can tentatively conclude only that our results are in agreement with these indications, it will be necessary to corroborate our comparative analyses results in future studies with different experimental setups before any conclusions about genetic and metabolic adaptations of these two species to environmental stress can be made with confidence. Regardless of this, our analyses enabled us to identify a number of genes (e.g. *AAT1*, *metE*, *pip*) and metabolites (e.g. rutin, raffinose, galactinol, trehalose, citrulline and DL-arginine) that may be of interest to future functional studies of stress adaptation in plants. In the light of the rapid growth in global popularity of “health food” and “organic food” products^71^, we expect that both of these species shall continue to receive increasing scientific attention. Additionally, *L. ruthenicum* has high suitability for combating soil desertification and for alleviating soil salinity/alkalinity^8,9^, which is a major problem both in China and globally^72,73^, and there are indications that it may also have a very high capacity for removal of petroleum from contaminated soil^74^. Therefore, this indicates that *L. ruthenicum* may have much higher potential for human use, than its current, highly localized, relevance appears to imply.

## Supplementary information

Supplementary Figures.

Supplementary Results.

Supplementary Dataset S1.

Supplementary Dataset S2.

Supplementary Dataset S3.

Supplementary Dataset S4.

Supplementary Dataset S5.

Supplementary Dataset S6.

## Data Availability

The datasets supporting the results of this article are available in the NCBIs Gene Expression Omnibus database (GEO) under the accession numbers GPL25820 (LB)^75^ and GPL25821 (LR)^76^.
