# Supplementary material for: Transcriptomic and metabolomic analyses of Lycium ruthenicum and Lycium barbarum fruits during ripening
Source: Sci Rep. 2020 Mar 9;10:4354. doi: 10.1038/s41598-020-61064-5 (PMC7062791; doi:10.1038/s41598-020-61064-5)
Supplement: Supplementary file 1 — Supplementary Figures. [file 41598_2020_61064_MOESM1_ESM.pdf]

Transcriptomic and metabolomic analyses of *Lycium ruthenicum* and *Lycium barbarum* fruits during ripening

Jianhua Zhao, Haoxia Li, Yue Yin, Wei An, Xiaoya Qin, Yajun Wang, Yanlong Li, Yunfang Fan, Youlong Cao

Supplementary Figures.

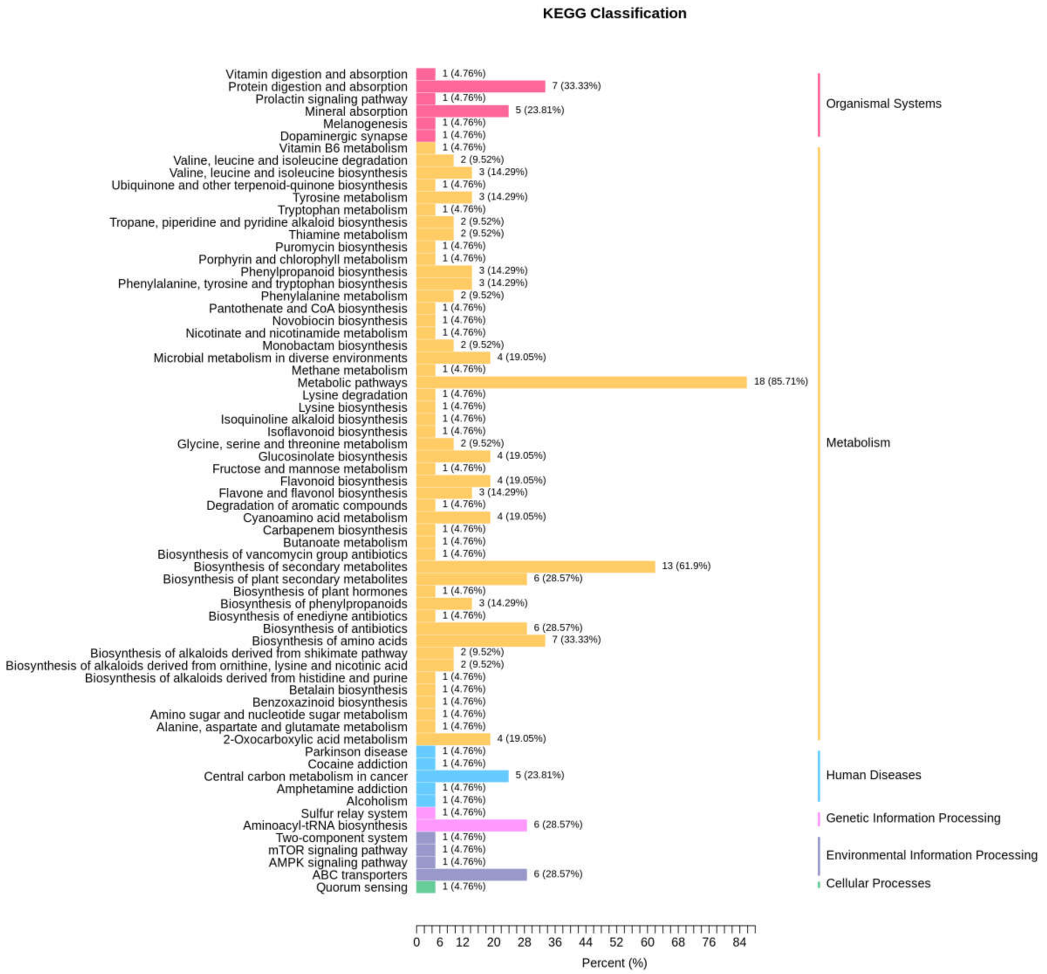

Figure S1. LB metabolomic data: pairwise interstage KEGG metabolic pathway enrichment: S1 vs. S2.

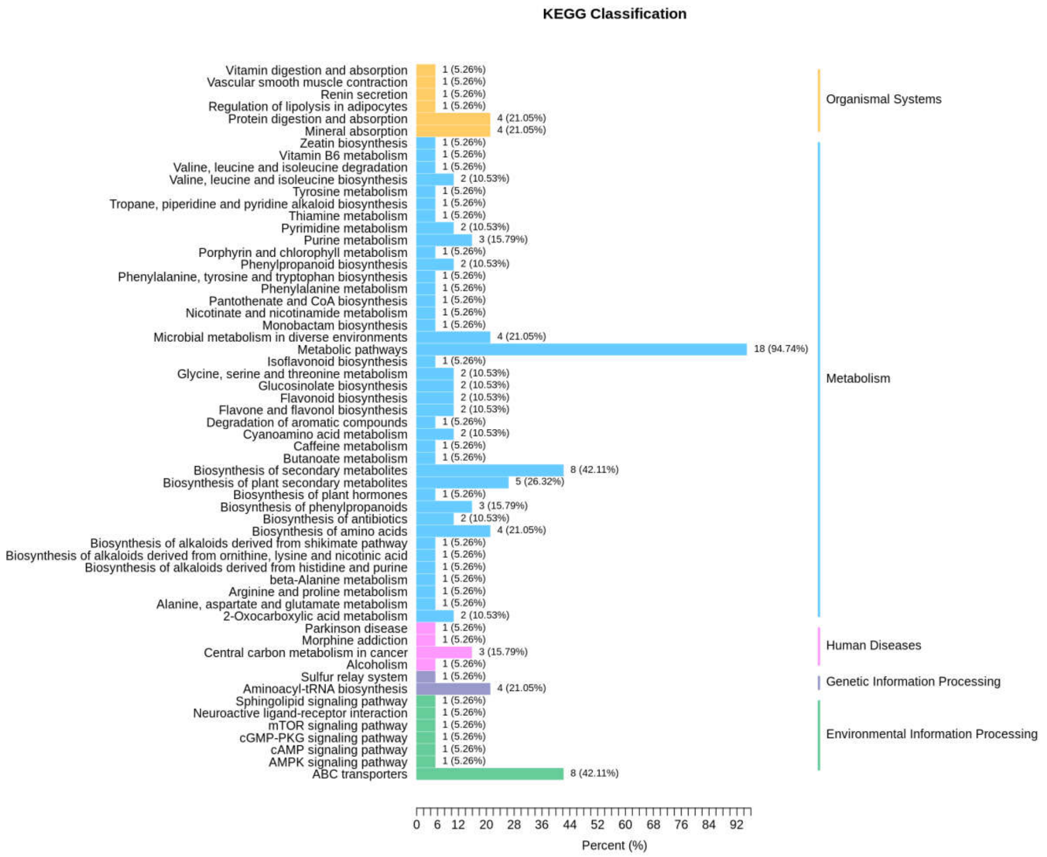

Figure S2. LB metabolomic data: pairwise interstage KEGG metabolic pathway enrichment: S2 vs. S3.

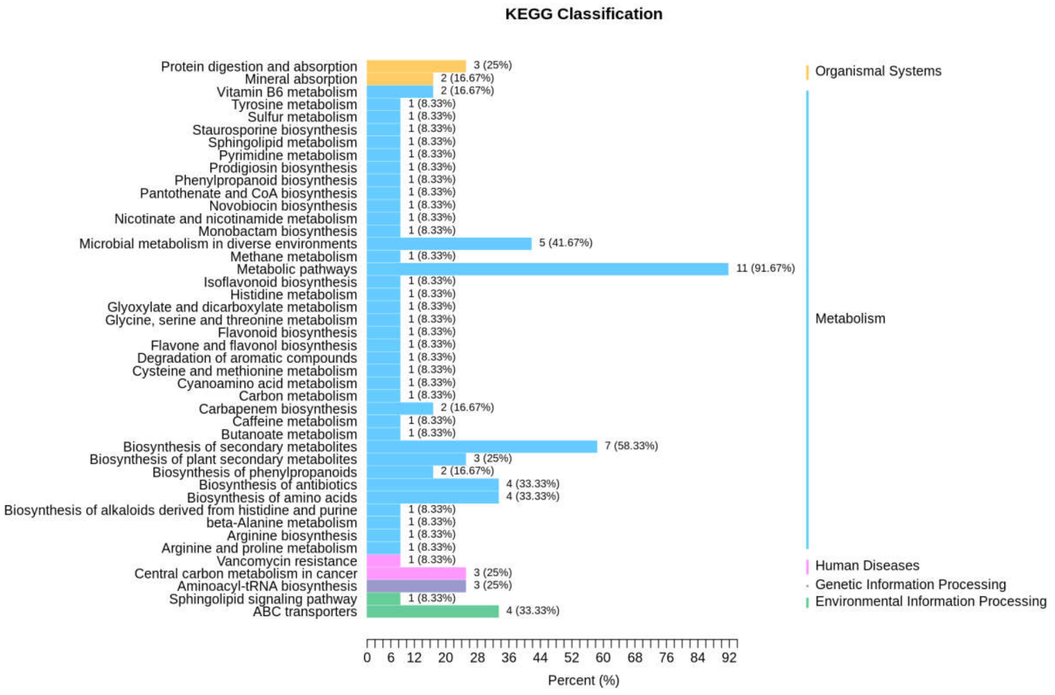

Figure S3. LB metabolomic data: pairwise interstage KEGG metabolic pathway enrichment: S3 vs. S4.

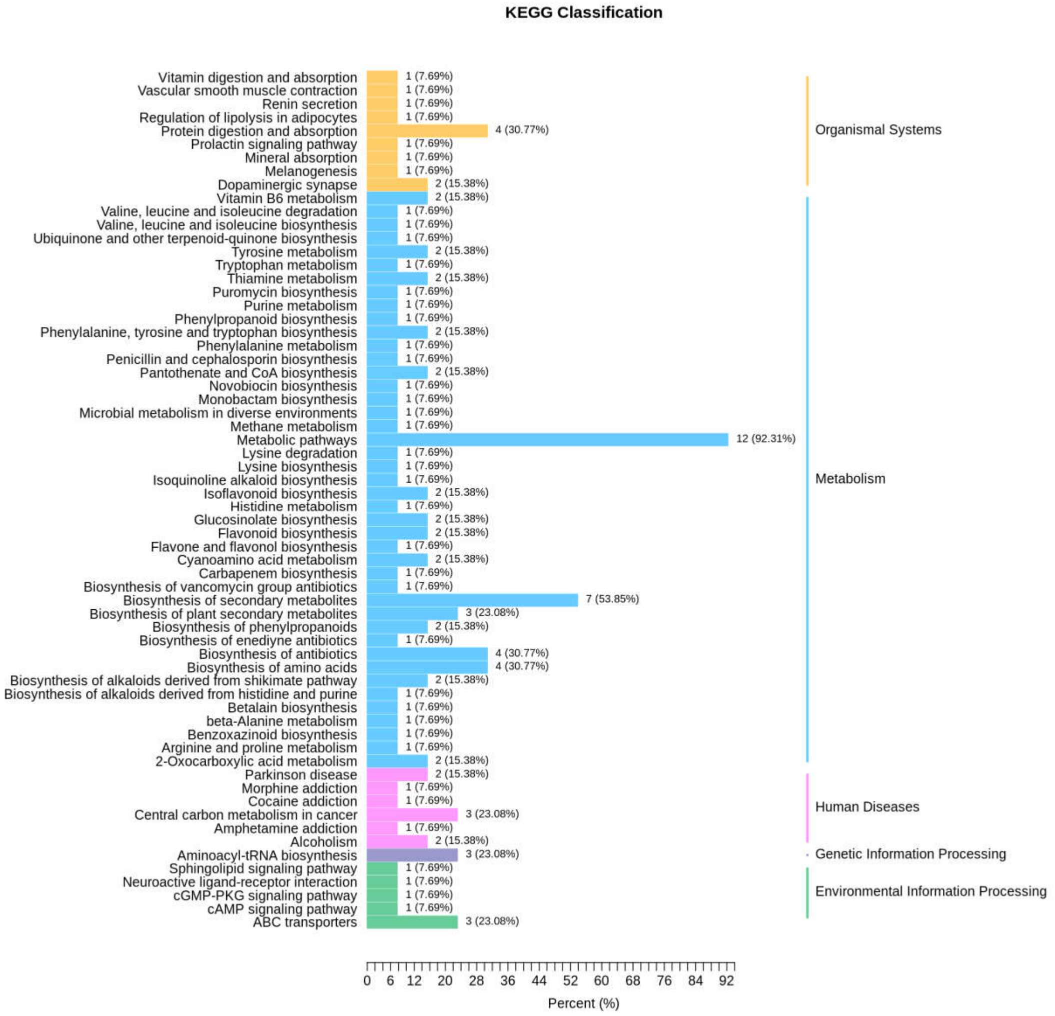

Figure S4. LB metabolomic data: pairwise interstage KEGG metabolic pathway enrichment: S4 vs. S5.

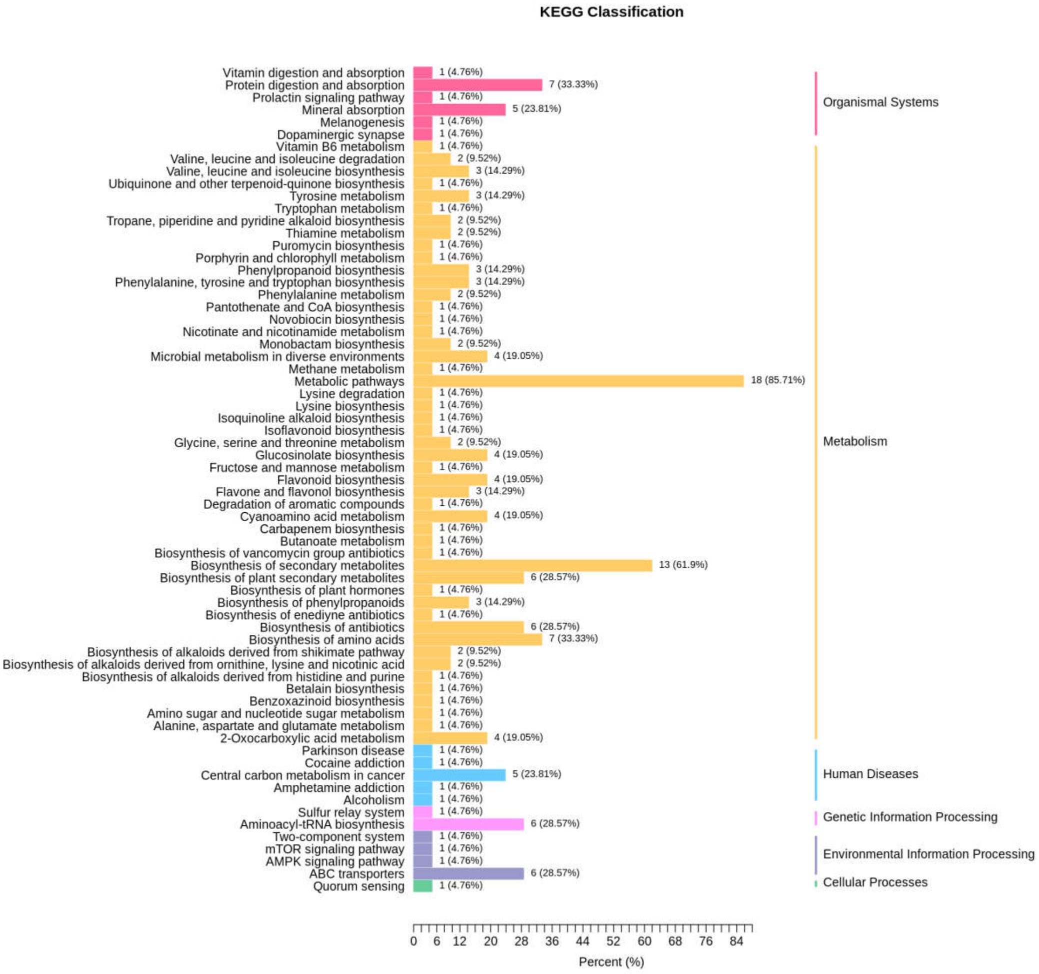

Figure S5. LR metabolomic data: pairwise interstage KEGG metabolic pathway enrichment: S1 vs. S2.

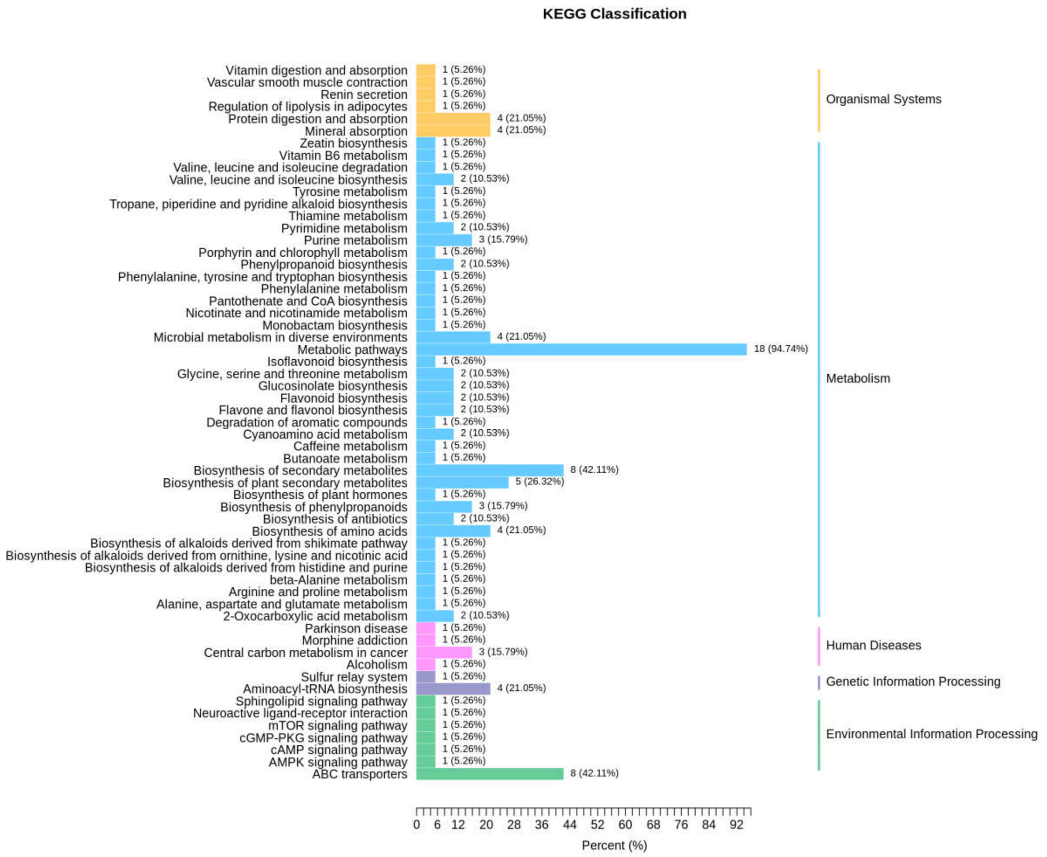

Figure S6. LR metabolomic data: pairwise interstage KEGG metabolic pathway enrichment: S2 vs. S3.

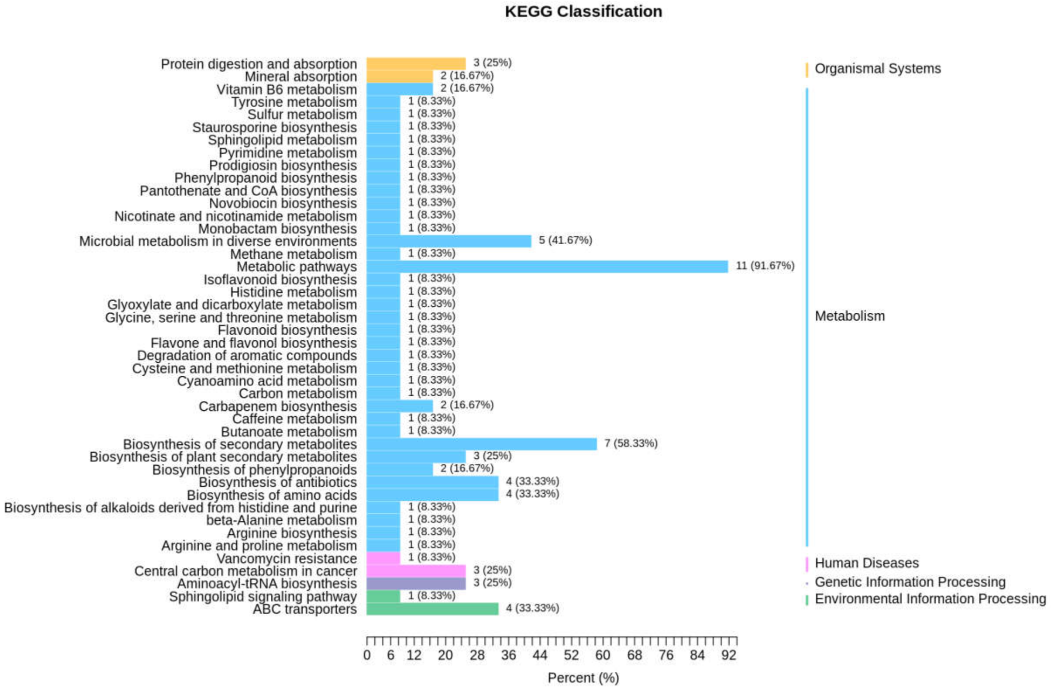

Figure S7. LR metabolomic data: pairwise interstage KEGG metabolic pathway enrichment: S3 vs. S4.

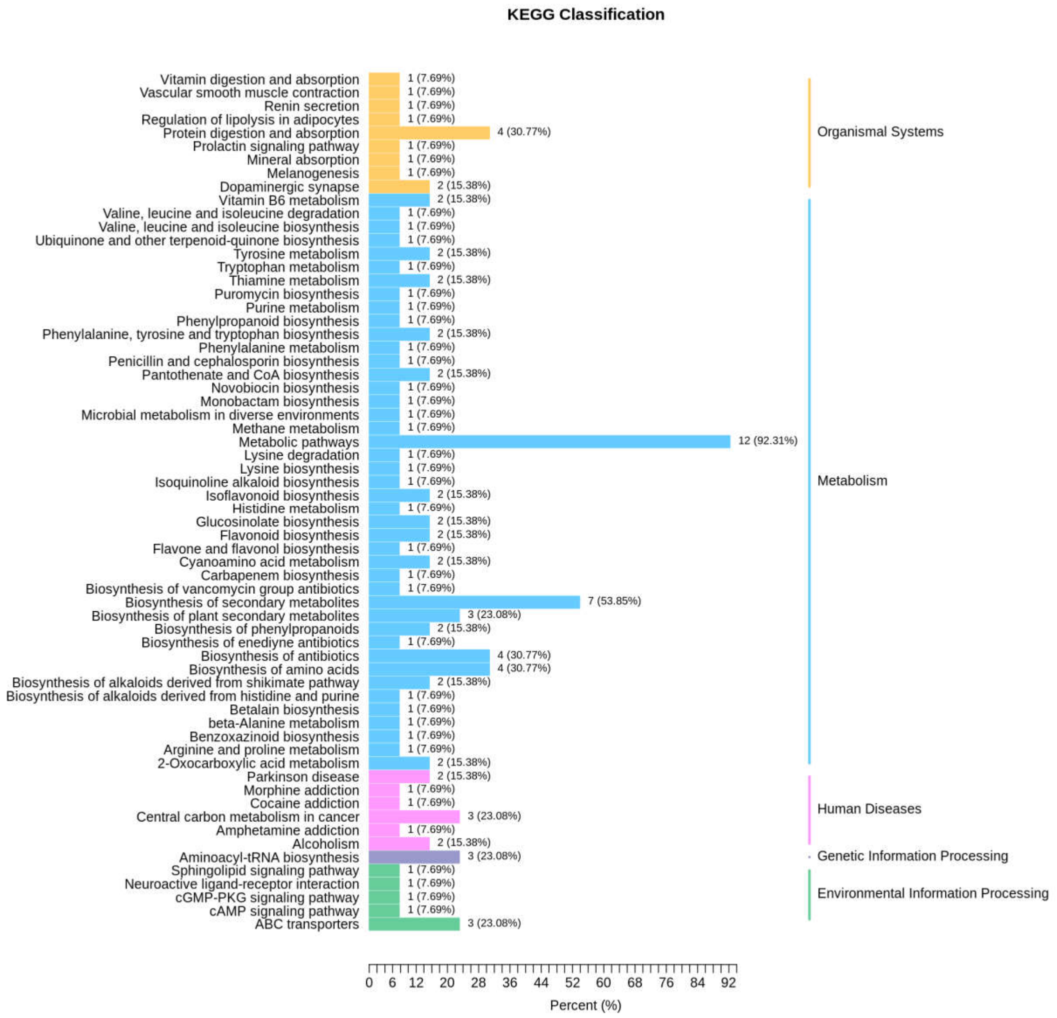

Figure S8. LR metabolomic data: pairwise interstage KEGG metabolic pathway enrichment: S4 vs. S5.

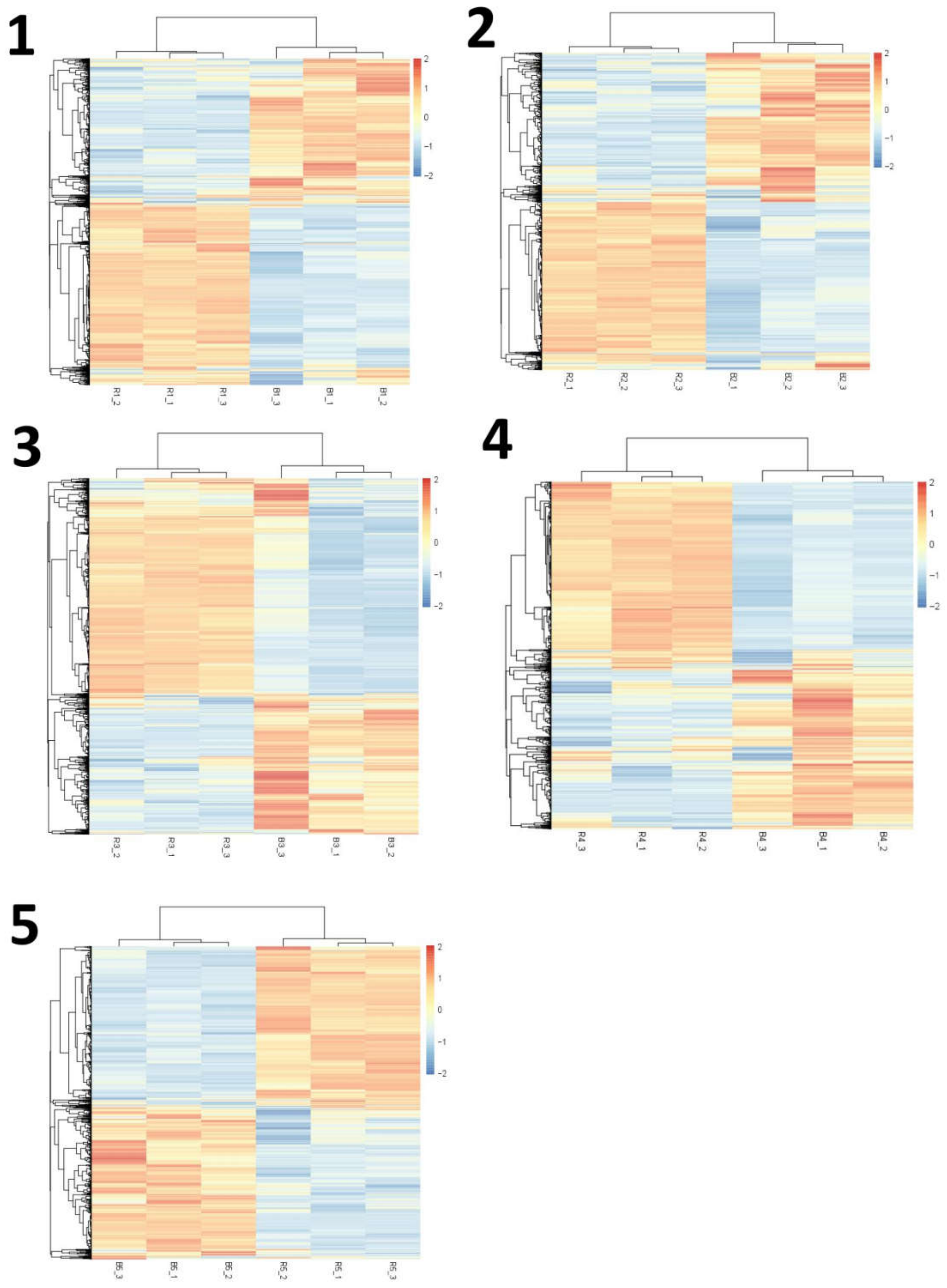

Figure S9. Pairwise comparison of differentially expressed genes (DEGS) between *L. barbarum* and *L. ruthenicum* during five sampled developmental stages. Developmental stage number (1-5) is indicated next to the corresponding heatmap. Samples are labelled B/R1-5_1-3, where the B stands for LR (black goji, *L. ruthenicum*) and R for LB (red goji, *L. barbarum*), followed by the developmental stage of fruit (1-5), and individual sample number (1-3). Samples were grouped by hierarchical clustering; dendrograms above and left of the heatmap indicate relatedness of samples and DEGs respectively.

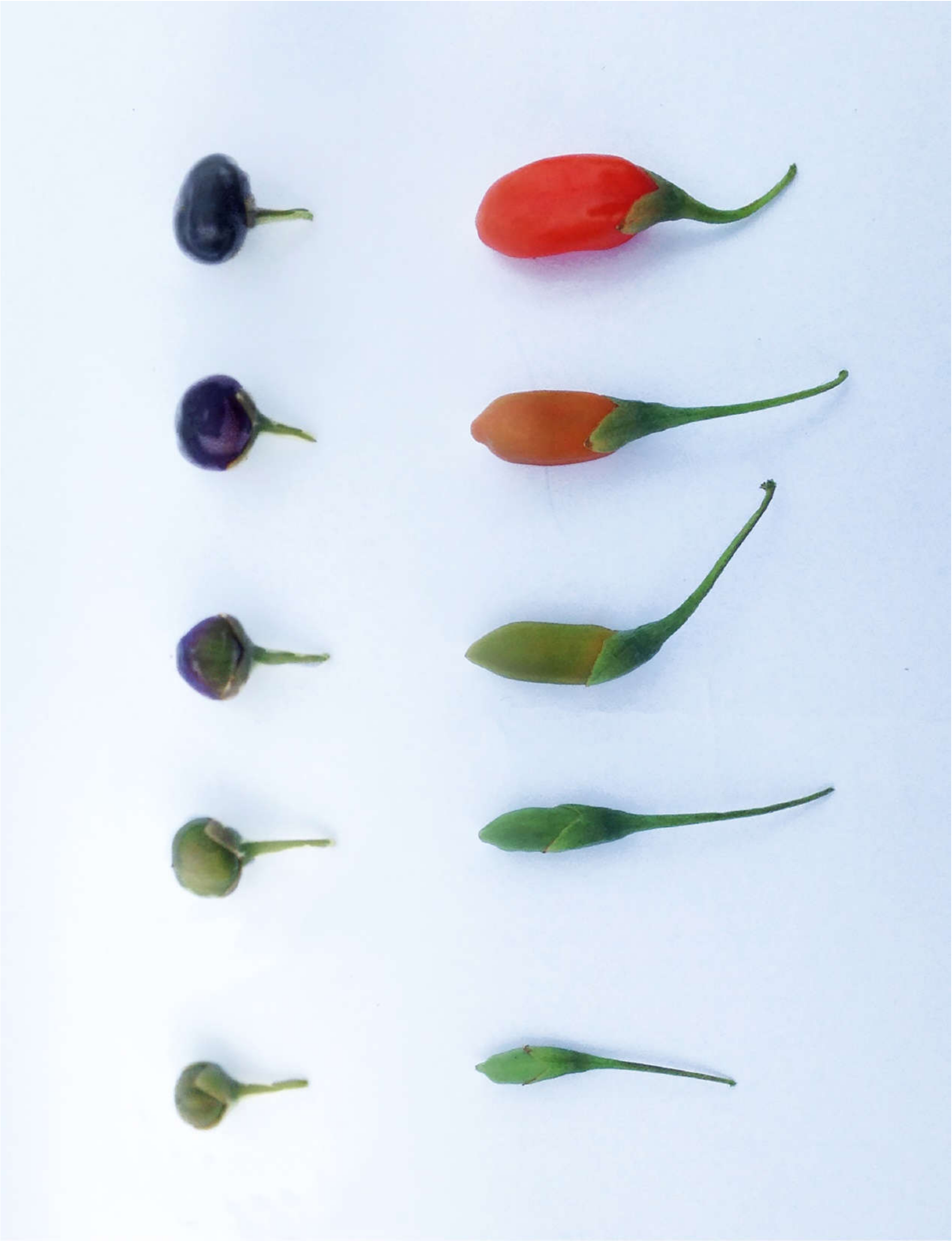

Figure S10. Fruits of LR (left) and LB (right) at five sample ripening stages (1 to 5, bottom to top).
