# Supplementary material for: Transcriptomic and metabolomic analyses of Lycium ruthenicum and Lycium barbarum fruits during ripening
Source: Sci Rep. 2020 Mar 9;10:4354. doi: 10.1038/s41598-020-61064-5 (PMC7062791; doi:10.1038/s41598-020-61064-5)
Supplement: Supplementary file 2 — Supplementary Results. [file 41598_2020_61064_MOESM2_ESM.pdf]

Transcriptomic and metabolomic analyses of *Lycium ruthenicum* and *L. barbarum* fruits during ripening

Jianhua Zhao, Haoxia Li, Yue Yin, Wei An, Xiaoya Qin, Yajun Wang, Yanlong Li, Yunfang Fan, Youlong Cao

Supplementary results: validation of DEGs by PCR.

Validation of DEGs by qPCR

To assess the reliability of the RNA-seq data, we performed quantitative real-time PCR (qPCR) for five randomly selected DEGs. We used the same RNA samples that were used for RNA-seq, and primers were designed using the sequences obtained from the transcriptome data. qPCR was performed on the Bio-Rad S1000 machine with Power SYBR Green PCR Master Mix (Applied Biosystems, MA, USA) following the manufacturers' instructions. The PCR conditions were 95°C for 3 min, followed by 40 cycles of 95°C for 10 s, 55°C for 20 s, and 72 °C for 20 s. PCR amplifications were performed in triplicate for each sample. The suitability of two commonly used reference genes, β-actin and 18s, was tested. Results indicate that both genes are sufficiently stable (see the end of the file), but as 18S was used as reference gene in *Lycium* species before (Ji et al. 2009), we also selected 18S. Expression levels of the target genes were normalized to *18S* (reference gene) and calculated using the $2^{-\Delta\Delta Ct}$ method (Livak and Schmittgen 2001) with the lowest-expression stage selected as the calibrator sample.

RNA-seq results for genes selected for qPCR confirmation. LR is *Lycium ruthenicum*, LB is *Lycium barbarum*.

**Gene ID**	**LB1_1**	**LB1_2**	**LB1_3**	**LB2_1**	**LB2_2**	**LB2_3**	**LB3_1**	**LB3_2**	**LB3_3**	**LB4_1**	**LB4_2**	**LB4_3**	**LB5_1**	**LB5_2**	**LB5_3**
DN94308_c2_g4	53.7	41.93	42.81	1.24	0.87	0.93	0.4	0.15	0.15	0.28	0.39	0.63	0.12	0	0.4
DN80120_c0_g1	43.5	44.65	48.8	304.57	474.57	418.93	1173.77	893.83	1079.26	1253.07	1346.18	527.93	1242.49	776.89	1078.25
DN94308_c2_g5	34.84	41.3	36.28	0.71	0.76	0	0	0	0	0	0.73	0	0	0	0
DN79928_c2_g1	403.83	492.86	368.35	25.08	32.53	20.27	38.01	51.92	35.43	12.9	25.25	14.42	40.19	29.29	26.45
DN75127_c1_g1	132.65	103.94	105.87	70.88	48.99	41.71	1.61	1.47	1.55	0.65	2.5	15.22	0.49	11.56	1.3
DN73389_c1_g2	35.72	32.52	54.58	50.3	35.88	39.51	11.63	15.41	27.87	6.46	30.88	1.94	41.22	29.55	47.24
DN68066_c0_g2	8.39	1.37	6.51	38.16	66.68	67.56	7.32	9.72	20.87	4.74	3.95	1.17	1.46	1.46	1
	**LR1_1**	**LR1_2**	**LR1_3**	**LR2_1**	**LR2_2**	**LR2_3**	**LR3_1**	**LR3_2**	**LR3_3**	**LR4_1**	**LR4_2**	**LR4_3**	**LR5_1**	**LR5_2**	**LR5_3**
DN94308_c2_g4	13.85	13.02	5.84	5.41	10.8	8.6	2.25	0.79	2.34	2.47	0.51	1.02	0	0	0.1
DN80120_c0_g1	4.83	4.64	5.14	12.88	7.81	35.19	87.09	112.61	244.15	284.47	241.18	198.02	841.93	972.11	741.48
DN94308_c2_g5	18.8	10.64	11.61	5.33	6.71	7.11	1.62	2.84	2.5	1.48	0	0.62	0	0.52	0
DN79928_c2_g1	2.91	0	2.23	5.06	2.24	1.02	12.53	11.04	13.06	23.8	30.41	25.72	7.86	9.5	24.15
DN75127_c1_g1	26.8	32.35	24.16	10.38	25.28	47.4	3.51	5.84	4.48	2.65	2.31	1.61	0.36	0.3	1.19
DN73389_c1_g2	15.44	10.28	6.74	26.93	9.84	13.98	151.22	99.11	505.19	684.45	655.12	886.16	2511.1	2462.1	5268.62
DN68066_c0_g2	21.34	8.42	7.49	42.4	18.45	28.37	214.47	169.47	525.58	920.52	895.39	942.12	2756.98	2357.81	3730.38

Primers used for qPCR.

Gene ID	Putative gene name	Primer name	Sequence (5'-3')	TM (˚C)	Amplicon (bp)
DN94308_c2_g4	*Alcohol dehydrogenase*	ZJH DN94308F	TCGGATCTCATGGTTGTCGA	58.53	141
		ZJH DN94308R	GTGCATTCCAGGCTTGTCAA	59.04	
DN80120_c0_g1	*Beta-glucosidase*	ZJH DN80120F	CCAAATCCACATAGTATAGGCCA	57.76	101
		ZJH DN80120R	TGGATCAAACGTCAAAGGCT	57.73	
DN94308_c2_g5	*Alcohol dehydrogenase*	ZJH DN94308-2F	GTTTGGGCGTGGACTCATTT	59.04	105
		ZJH DN94308-2R	GAGGGTGAGTTGCAGAGACT	59.03	
DN79928_c2_g1	*Alcohol dehydrogenase*	ZJH DN79928F	CCATTGGCACGACTTCAACA	59.05	137
		ZJH DN79928R	GTTGCCTGTATTTCCCCTGC	59.18	
DN75127_c1_g1	*Peroxidase*	ZJH DN75127F	GTCATCAGGGTGTTGGAGGA	59.02	118
		ZJH DN75127R	ATAGCATCCCTAGCAGCGAG	59.11	
DN73389_c1_g2	*Chalcone synthase*	ZJH DN73389F	CATTCGAGCCCTTCACCAGT	60.04	153
		ZJH DN73389R	GTTGGCCCTAAAACCGGAGA	59.96	
DN68066_c0_g2	*Leucoanthocyanidin dioxygenase*	ZJH DN68066F	ACCTCCGGCAACCTTAACAC	60.25	172
		ZJH DN68066R	AGGACCTCAAGTACCGACGA	59.96	
Reference	*18S*	GouQi 18SF	AAAGGAATTGACGGAAGGGC	58.45	155
		GouQi 18SR	CAACTAAGAACGGCCATGCA	58.45	

qPCR results. Data are presented as mean values of triplicate samples ± SD. LR is *Lycium ruthenicum*, LB is *Lycium barbarum*.

DN94308_c2_g4

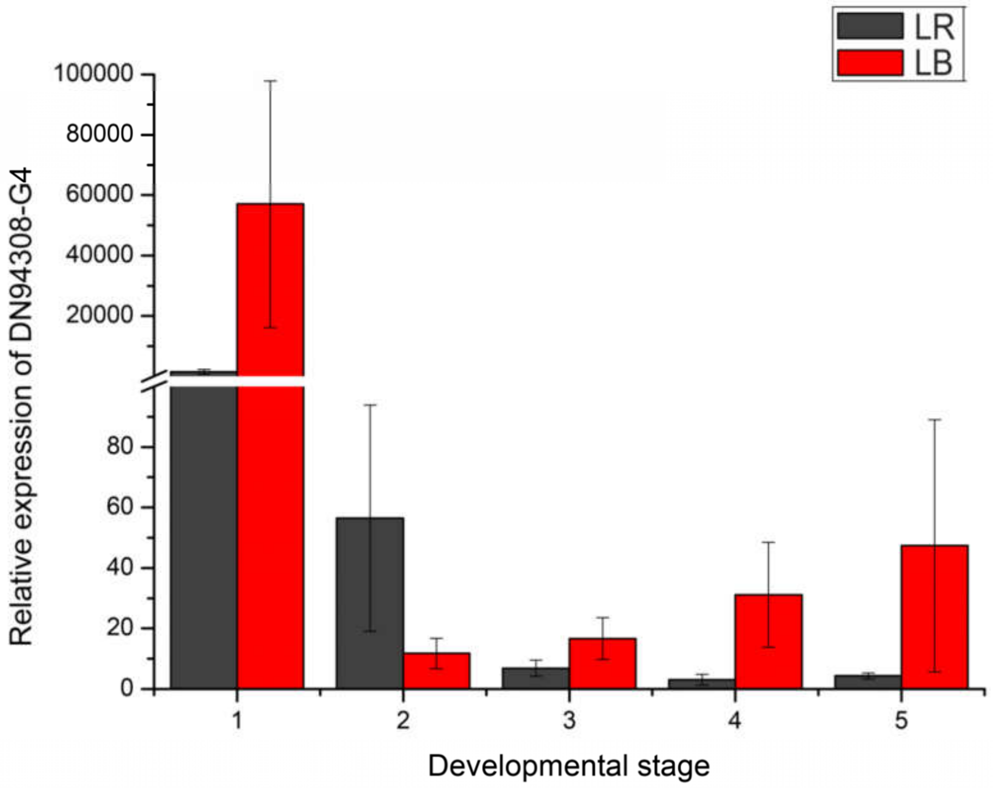

DN80120_c0_g1

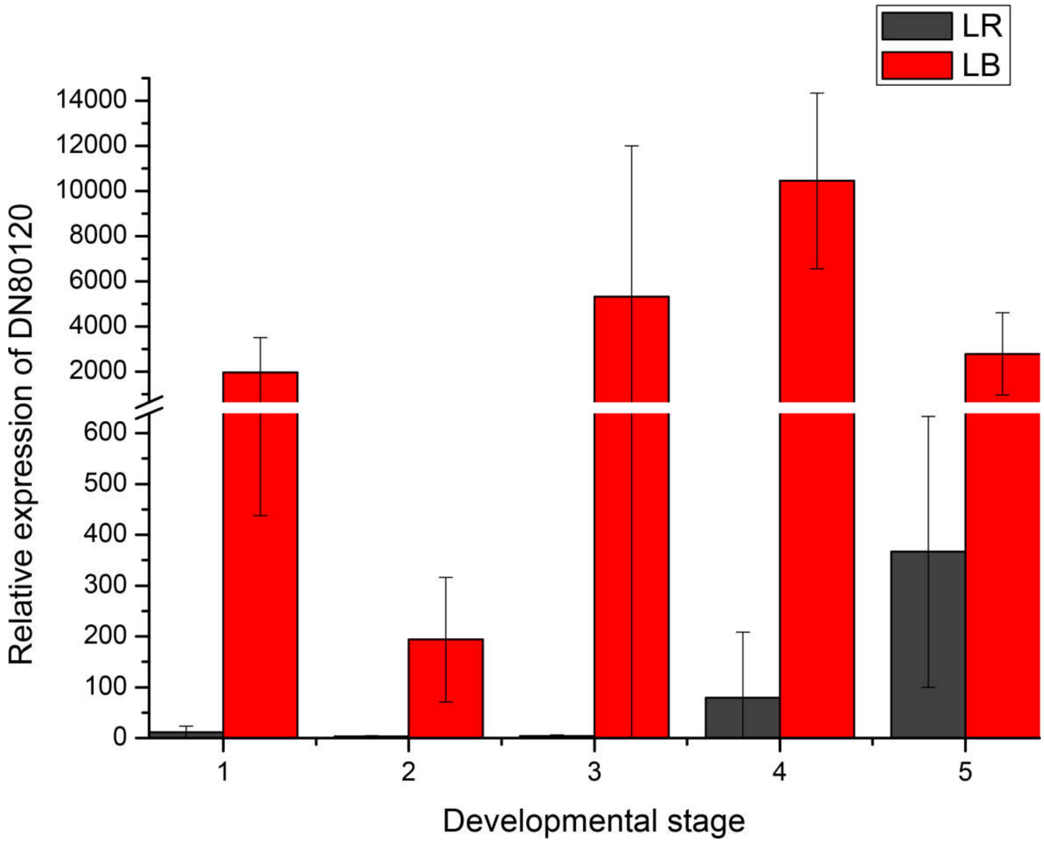

DN94308_c2_g5

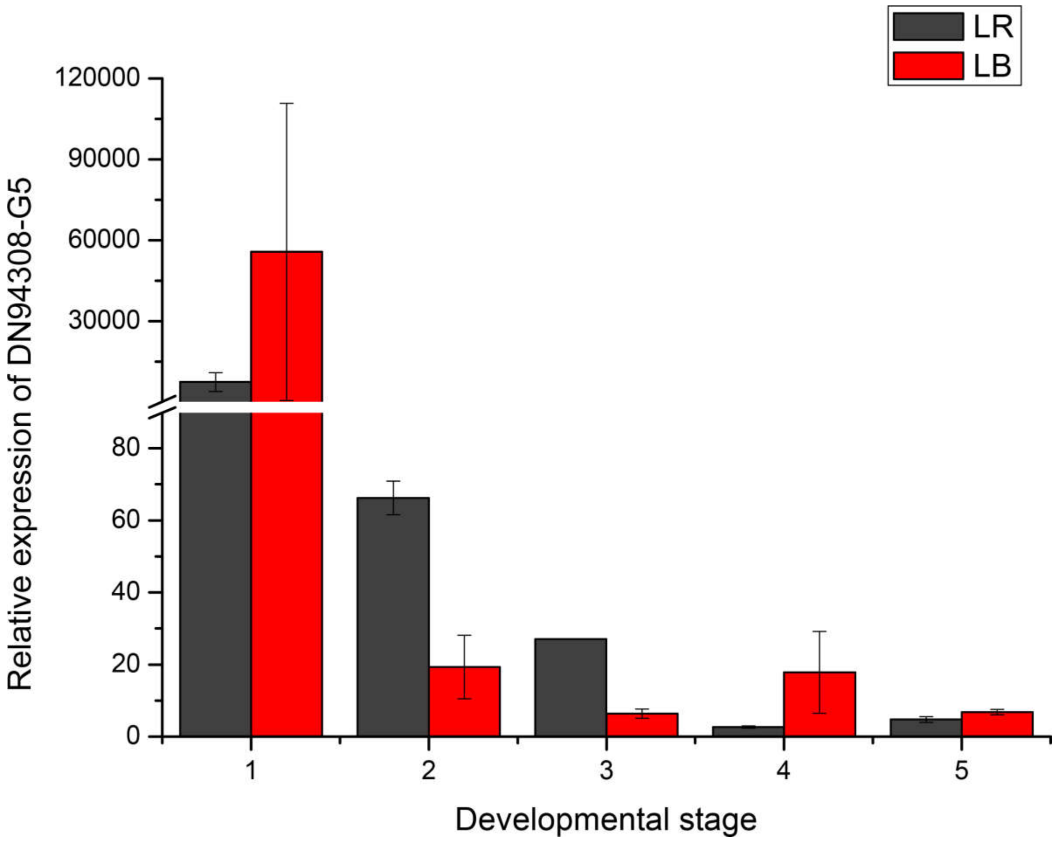

DN79928_c2_g1

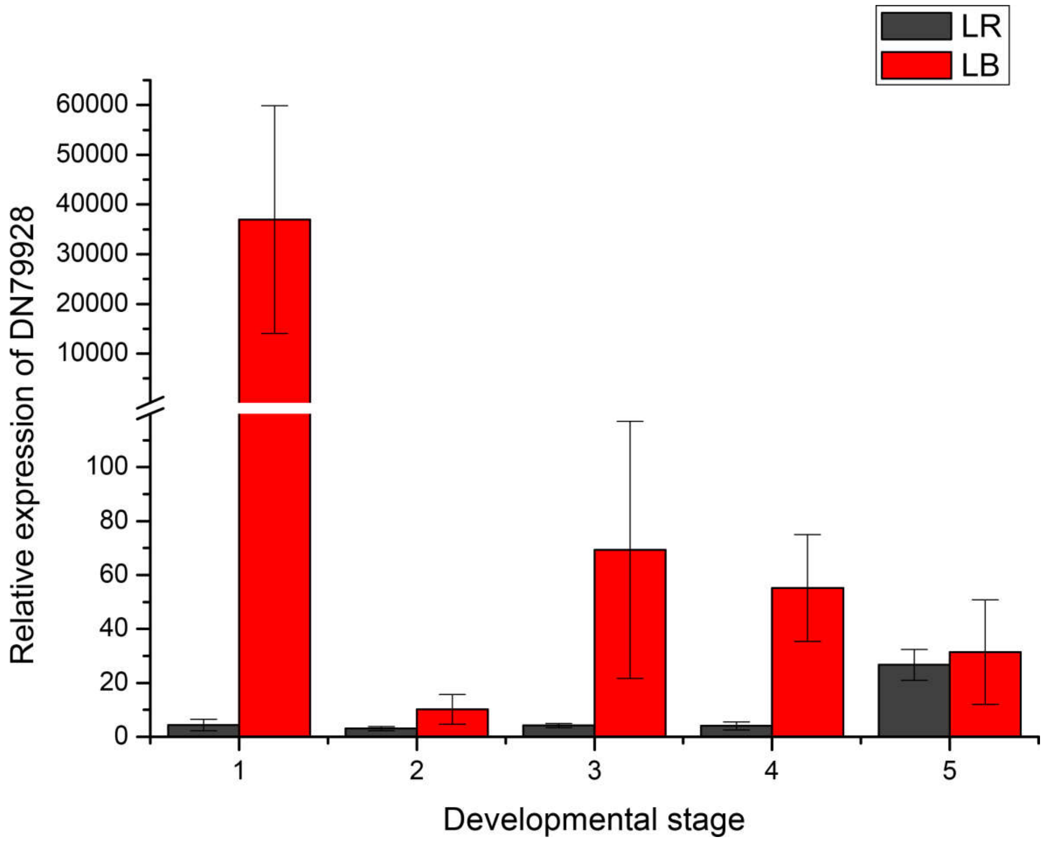

DN75127_c1_g1

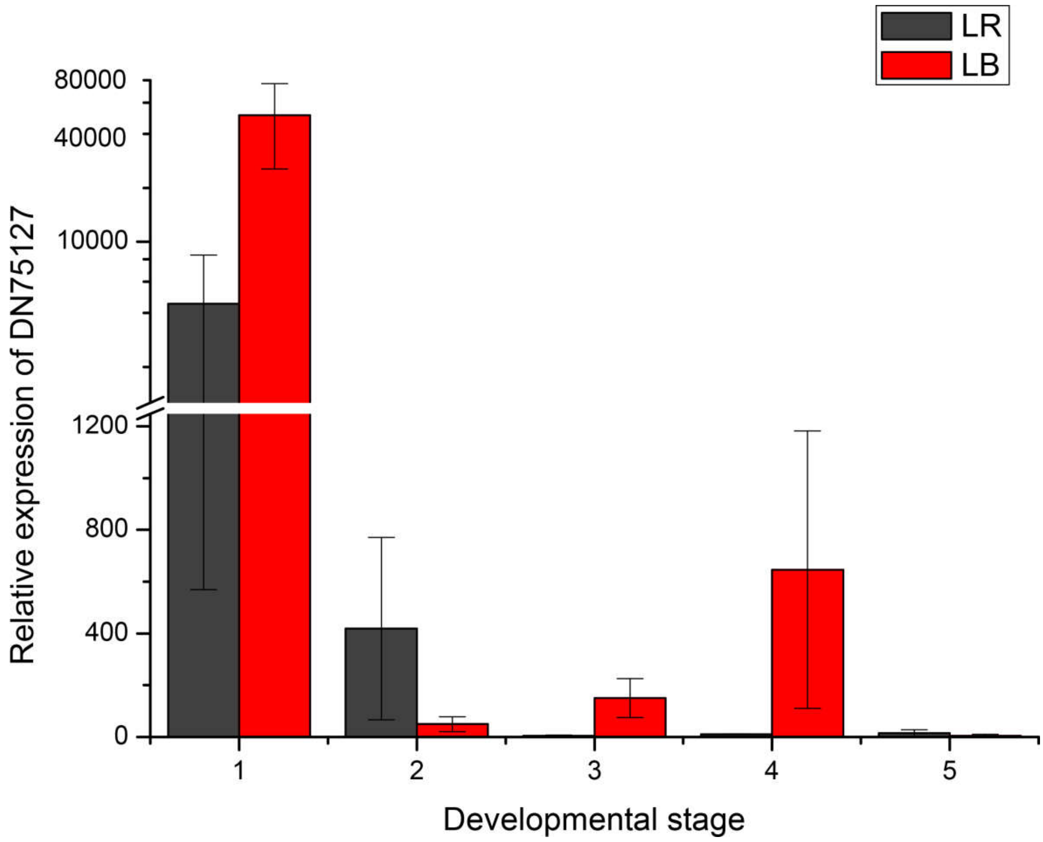

DN73389_c1_g2

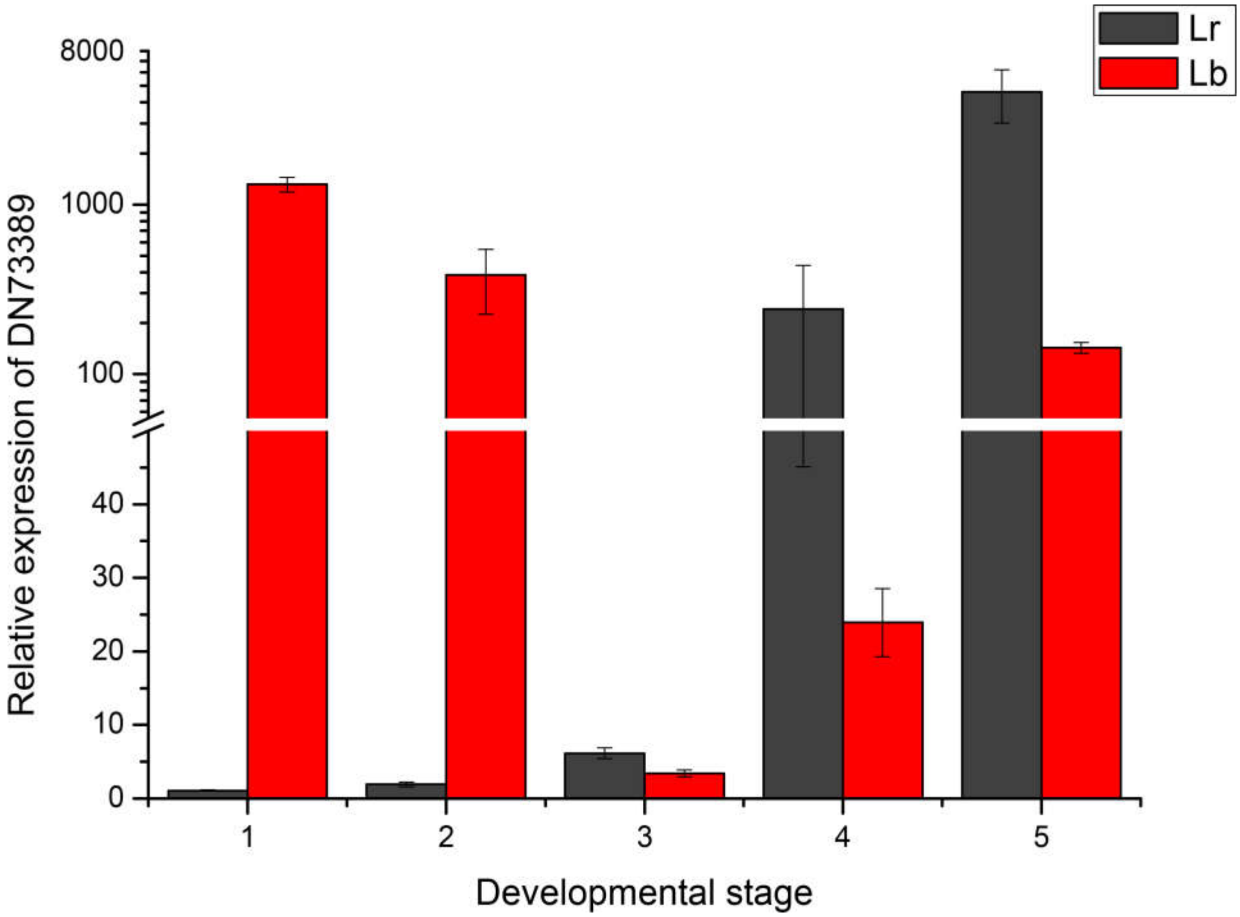

DN68066_c0_g2

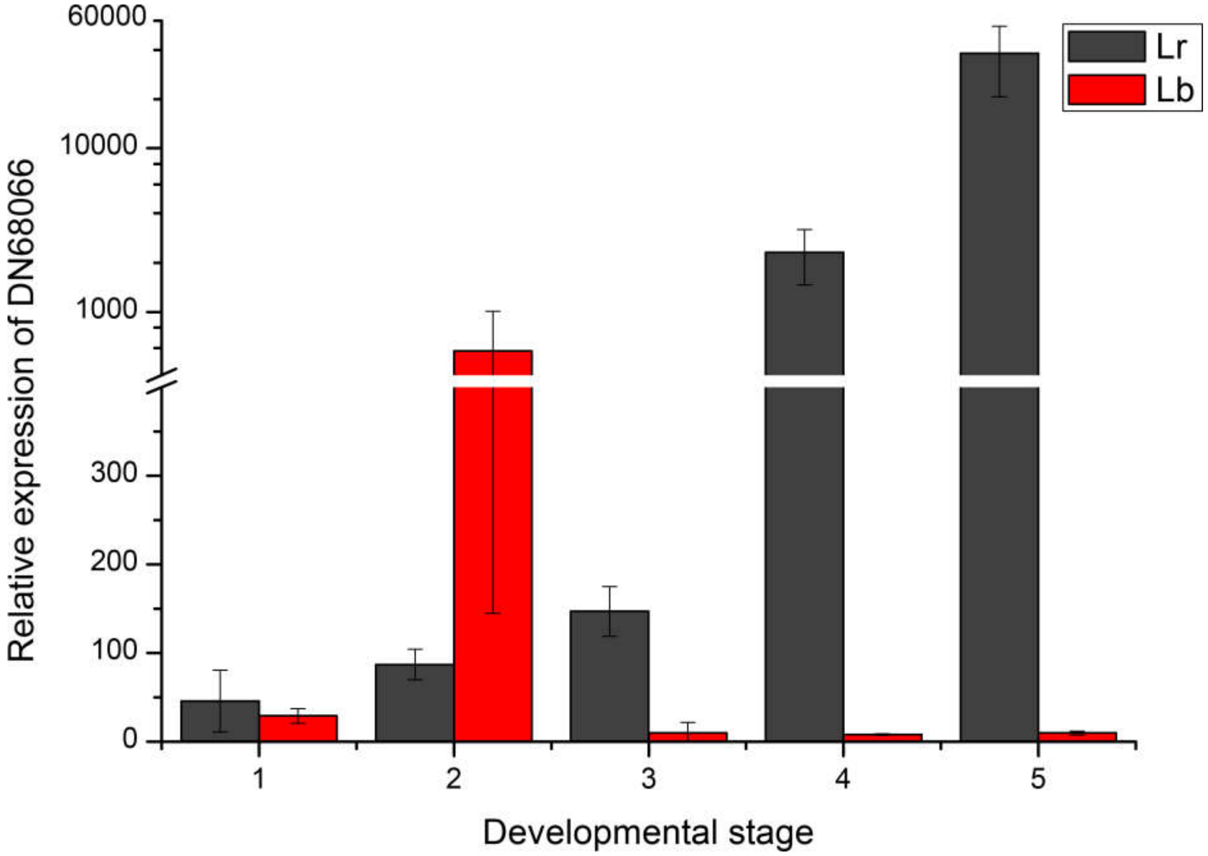

Melt curves

DN94308

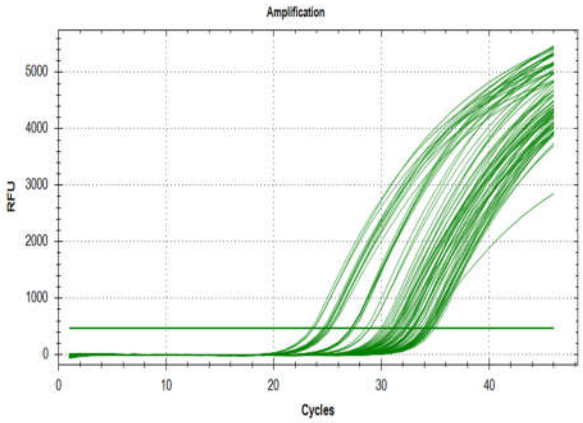

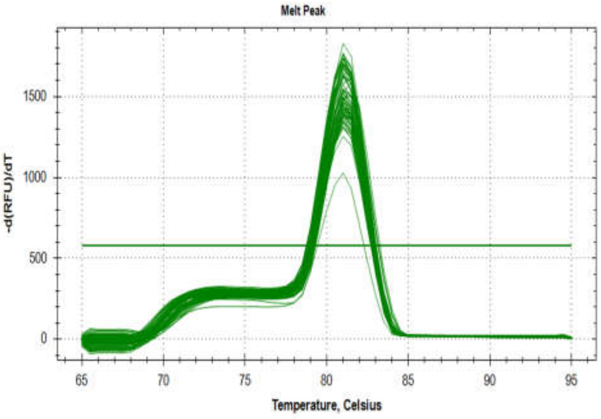

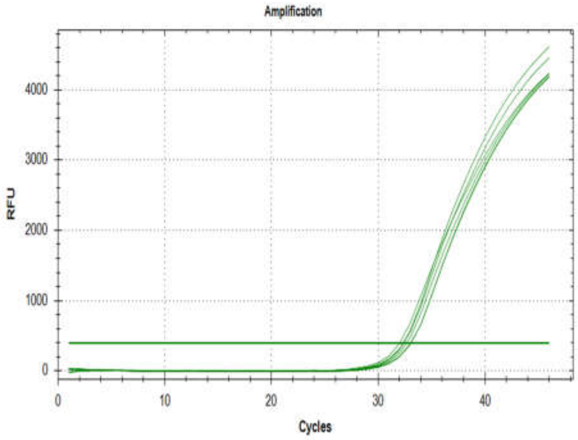

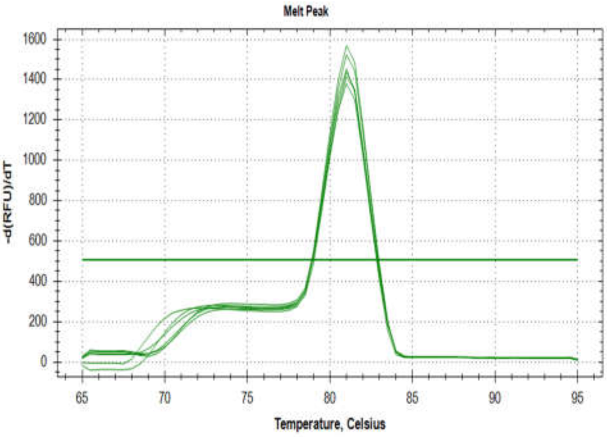

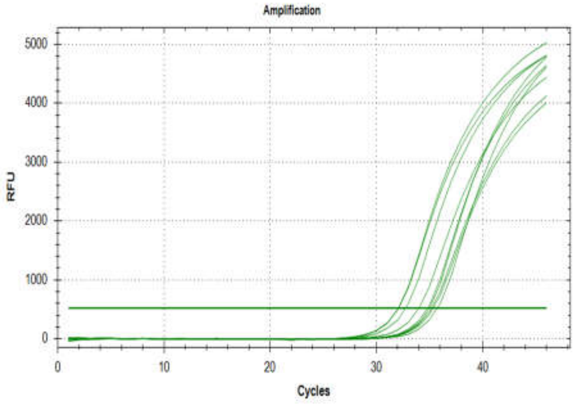

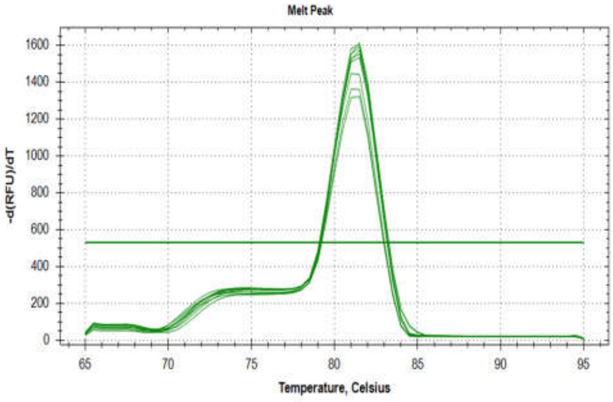

DN80120

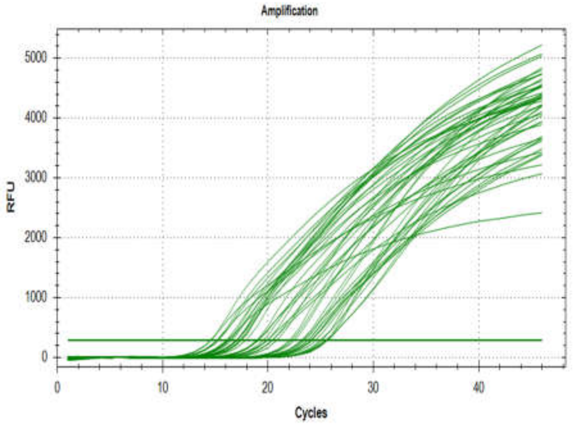
Amplification
RFU
Cycles
0
1000
2000
3000
4000
5000
0
10
20
30
40

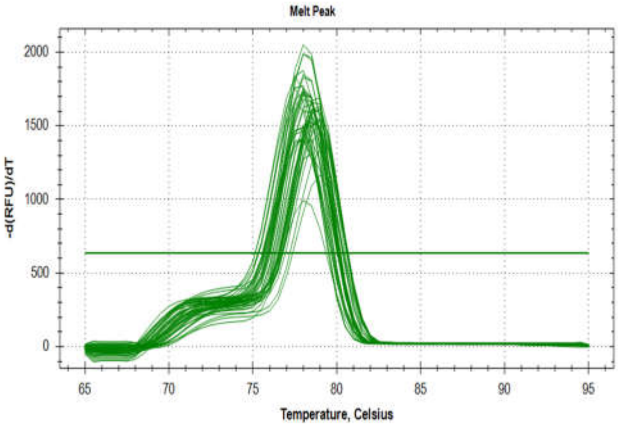
Melt Peak
-d(RFU)/dT
Temperature, Celsius
0
500
1000
1500
2000
65
70
75
80
85
90
95

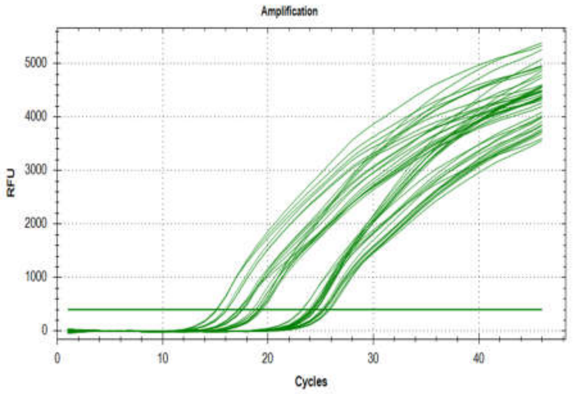
Amplification
RFU
Cycles
0
1000
2000
3000
4000
5000
0
10
20
30
40

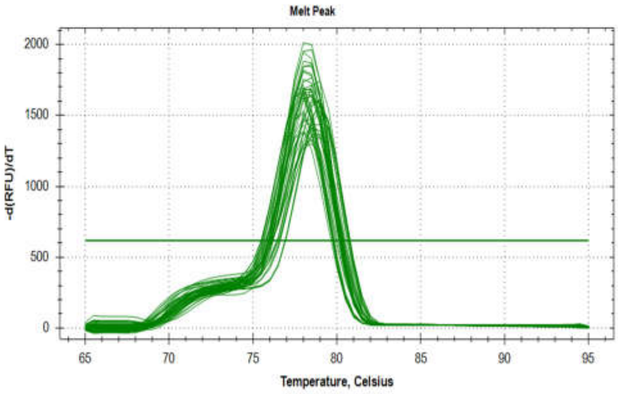
Melt Peak
-d(RFU)/dT
Temperature, Celsius
0
500
1000
1500
2000
65
70
75
80
85
90
95

DN94308

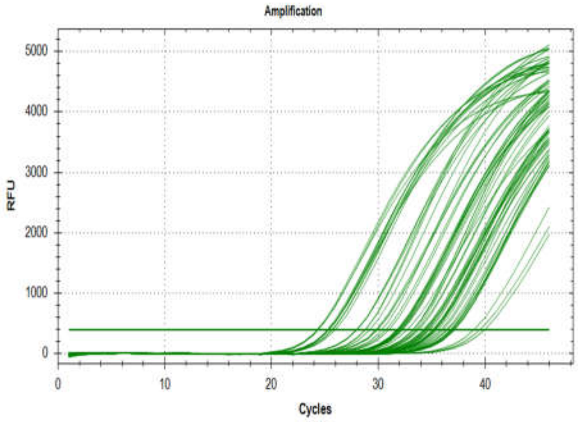
Amplification
5000
4000
3000
2000
1000
0
RFU
0
10
20
30
40
Cycles

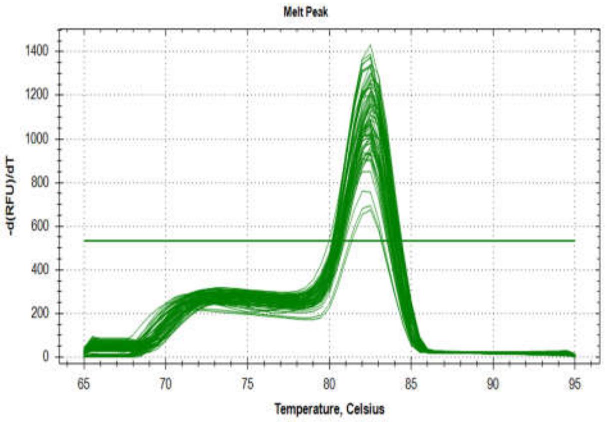
Melt Peak
1400
1200
1000
800
600
400
200
0
-d(RFU)/dT
65
70
75
80
85
90
95
Temperature, Celsius

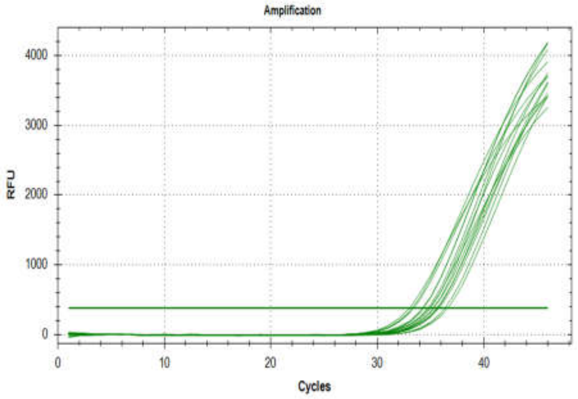
Amplification
4000
3000
2000
1000
0
RFU
0
10
20
30
40
Cycles

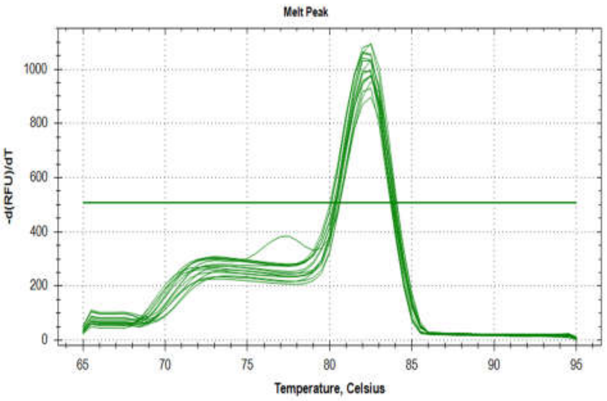
Melt Peak
1000
800
600
400
200
0
-d(RFU)/dT
65
70
75
80
85
90
95
Temperature, Celsius

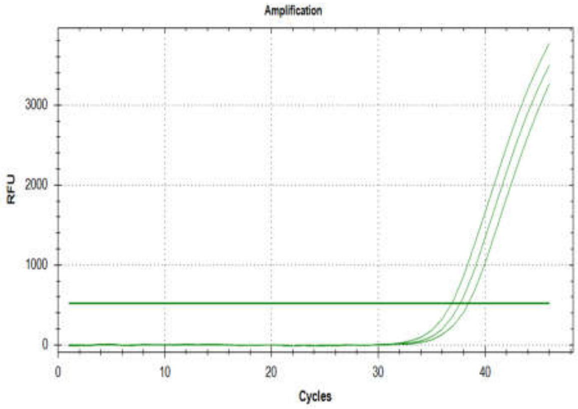
Amplification
3000
2000
1000
0
RFU
0
10
20
30
40
Cycles

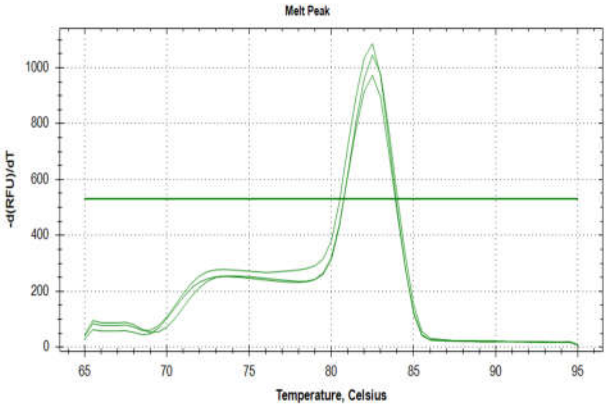
Melt Peak
1000
800
600
400
200
0
-d(RFU)/dT
65
70
75
80
85
90
95
Temperature, Celsius

DN79928

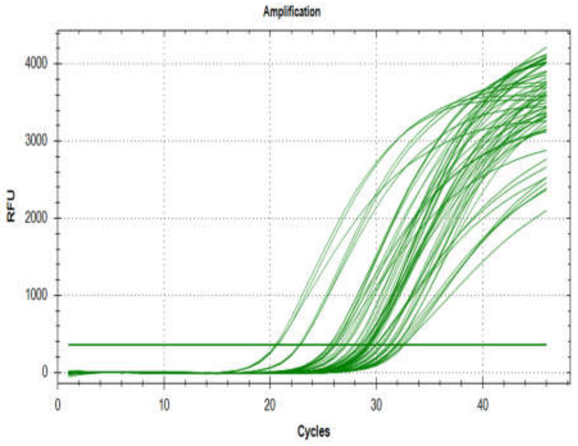

Amplification
RFU
Cycles

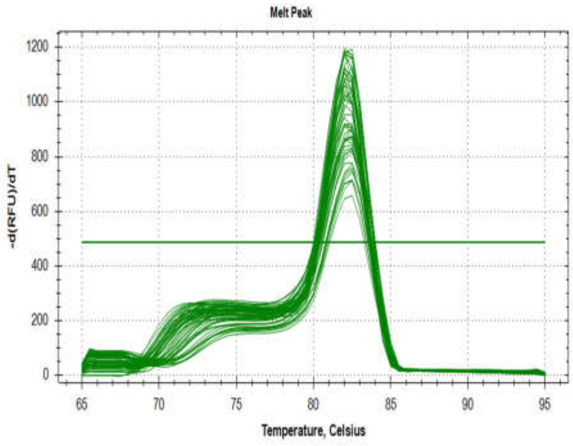

Melt Peak
-d(RFU)/dT
Temperature, Celsius

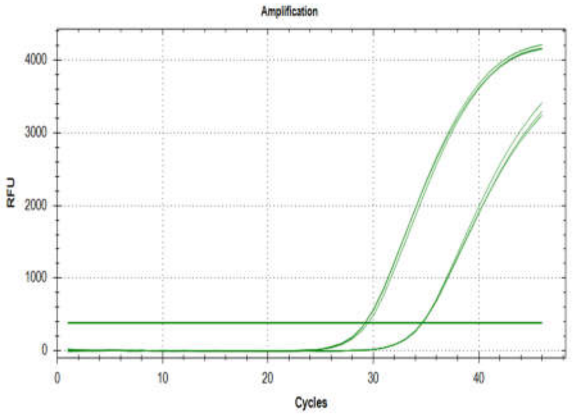

Amplification
RFU
Cycles

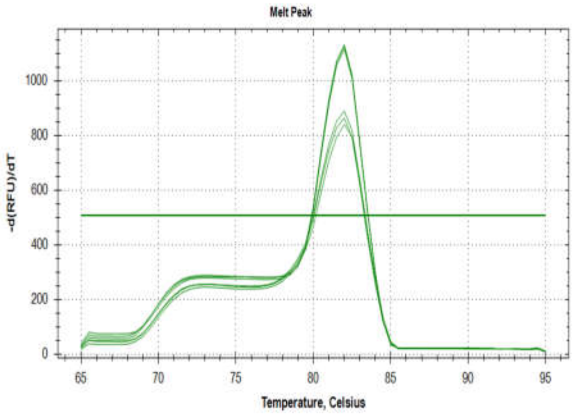

Melt Peak
-d(RFU)/dT
Temperature, Celsius

DN75127

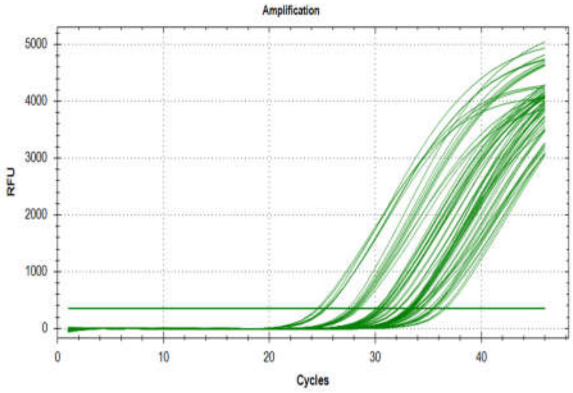
Amplification
RFU
5000
4000
3000
2000
1000
0
0
10
20
30
40
Cycles

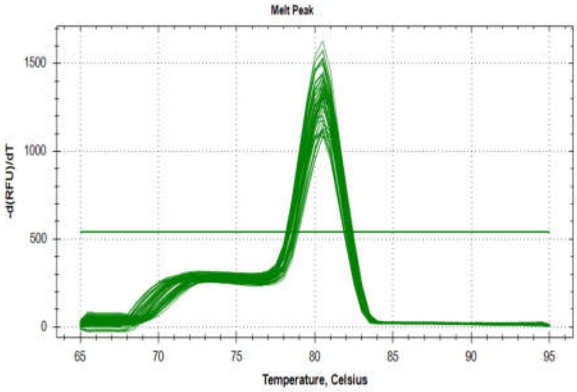
Melt Peak
-d(RFU)/dT
1500
1000
500
0
65
70
75
80
85
90
95
Temperature, Celsius

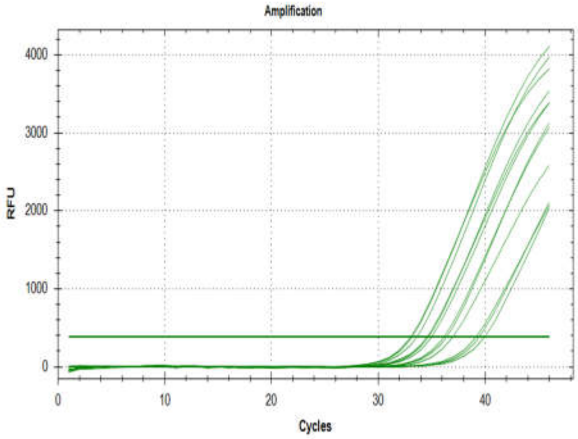
Amplification
RFU
4000
3000
2000
1000
0
0
10
20
30
40
Cycles

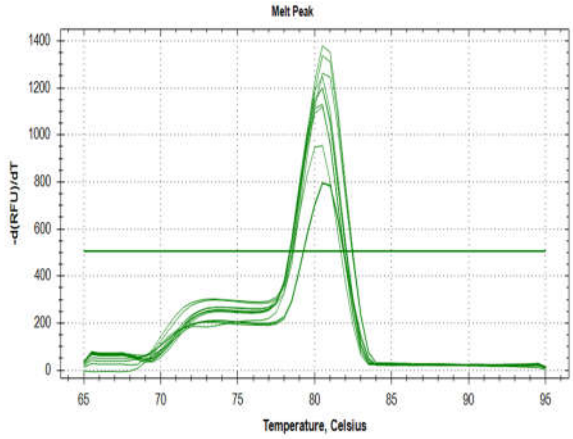
Melt Peak
-d(RFU)/dT
1400
1200
1000
800
600
400
200
0
65
70
75
80
85
90
95
Temperature, Celsius

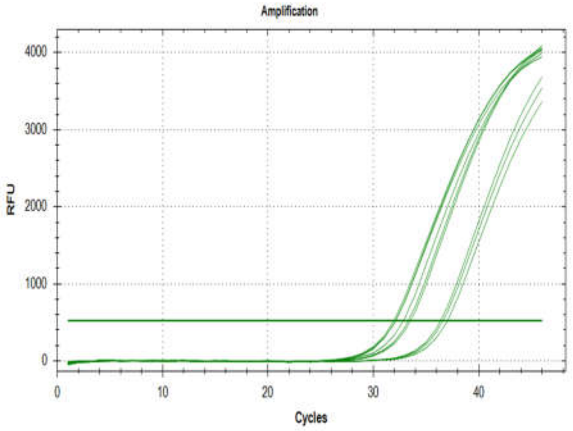
Amplification
RFU
4000
3000
2000
1000
0
0
10
20
30
40
Cycles

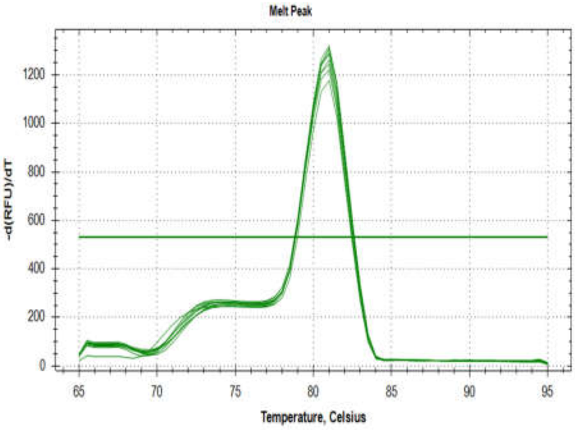
Melt Peak
-d(RFU)/dT
1200
1000
800
600
400
200
0
65
70
75
80
85
90
95
Temperature, Celsius

DN73389

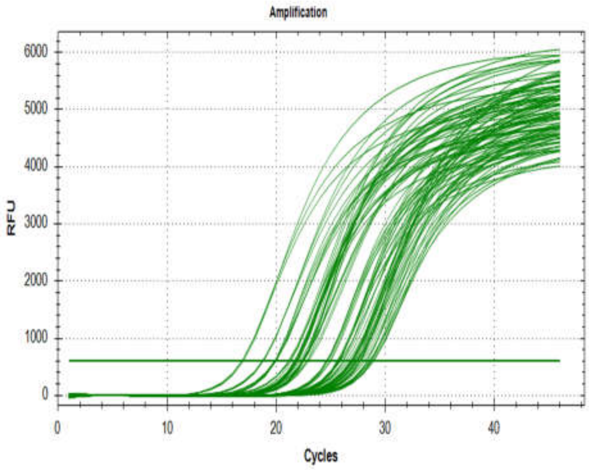

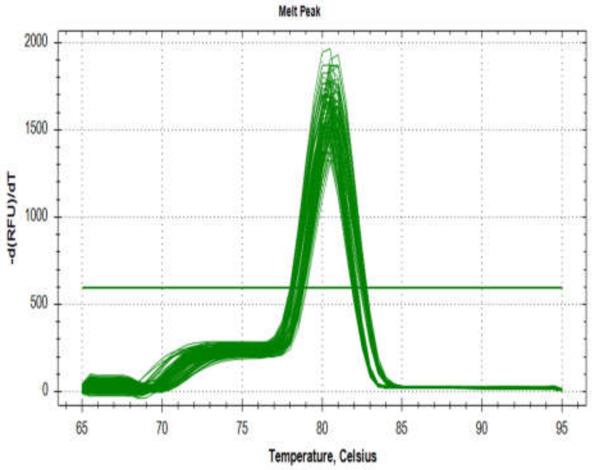

DN68066

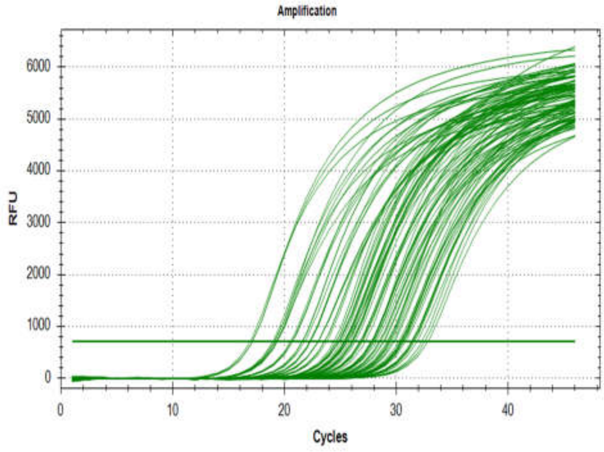

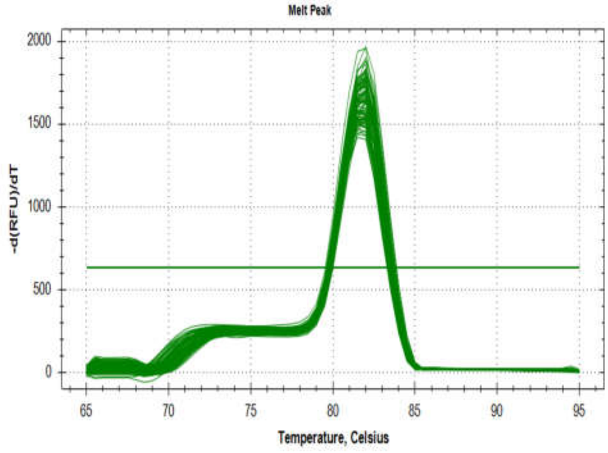

18S

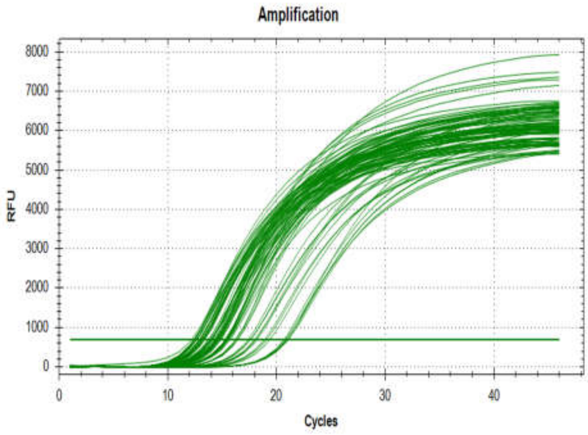

Amplification
8000
7000
6000
5000
4000
3000
2000
1000
0
RFU
0
10
20
30
40
Cycles

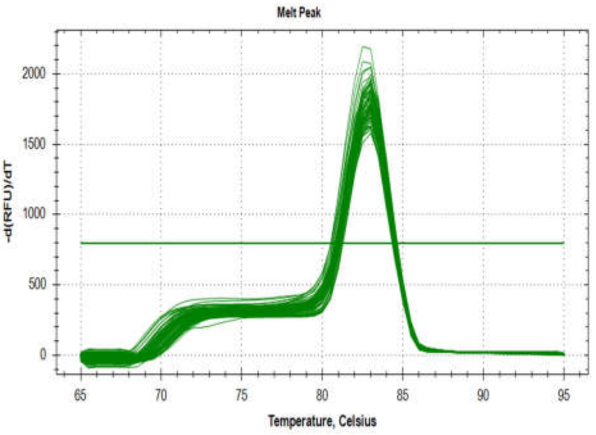

Melt Peak
2000
1500
1000
500
0
-d(RFU)/dT
65
70
75
80
85
90
95
Temperature, Celsius

Reference gene testing and comparison

18s

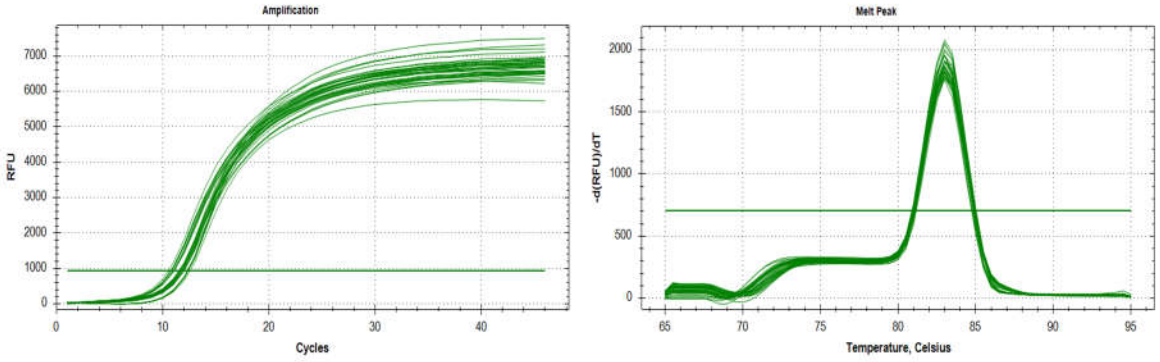

β-actin

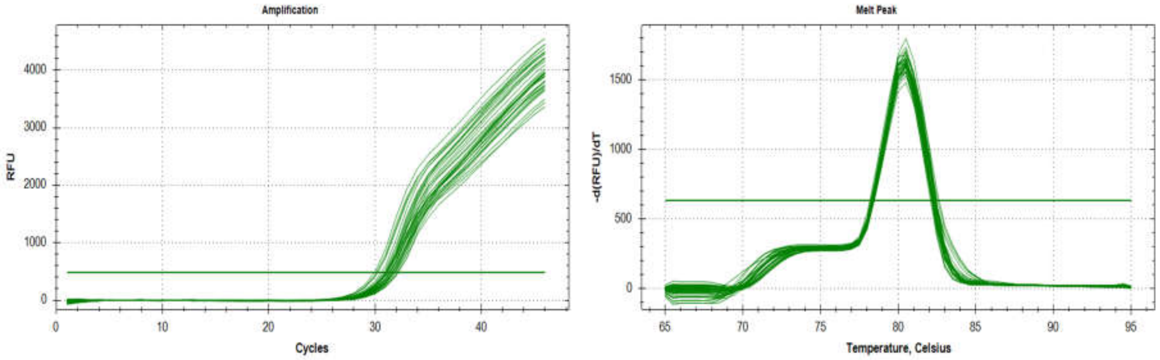

Primers

Gene	Name	Sequence 5’-3’	TM (℃)	Amplicon bp
18s	18S F	AAAGGAATTGACGGAAGGGC	58.45	155
	18S R	CAACTAAGAACGGCCATGCA	58.84	
β-actin	Actin F	TGTTGGTGACGAAGCTCAGT	59.54	160
	Actin R	AGTTAGGAGGACGGGATGCT	60.03	

References

Ji, Jing, Gang Wang, Jiehua Wang, and Ping Wang. 2009. “Functional Analysis of Multiple Carotenogenic Genes from Lycium Barbarum and Gentiana Lutea L. for Their Effects on β-Carotene Production in Transgenic Tobacco.” *Biotechnology Letters* 31 (2): 305–12. https://doi.org/10.1007/s10529-008-9861-8.

Livak, K J, and T D Schmittgen. 2001. “Analysis of Relative Gene Expression Data Using Real-Time Quantitative PCR and The2-DDCT Method.” *Methods* 25: 402–8. https://doi.org/10.1006/meth.2001.1262.
